# TCOF1 upregulation in triple-negative breast cancer promotes stemness and tumour growth and correlates with poor prognosis

**DOI:** 10.1038/s41416-021-01596-3

**Published:** 2021-10-30

**Authors:** Jianyang Hu, Yuni Lai, Hao Huang, Saravanan Ramakrishnan, Yilin Pan, Victor W. S. Ma, Wah Cheuk, Grace Y. K. So, Qingling He, C. Geoffrey Lau, Liang Zhang, William C. S. Cho, Kui Ming Chan, Xin Wang, Y. Rebecca Chin

**Affiliations:** 1 Tung Biomedical Sciences Centre, Department of Biomedical Sciences, City University of Hong Kong, Kowloon, Hong Kong; 2 City University of Hong Kong Shenzhen Research Institute, Shenzhen, China; 3 Department of Clinical Oncology, Queen Elizabeth Hospital, Kowloon, Hong Kong; 4 Department of Pathology, Queen Elizabeth Hospital, Kowloon, Hong Kong; 5 Department of Neuroscience, City University of Hong Kong, Kowloon, Hong Kong

**Keywords:** Breast cancer, Cancer stem cells

## Abstract

**Background:**

Triple-negative breast cancer (TNBC) is an aggressive subtype of breast cancer with poor prognosis. By performing multiomic profiling, we recently uncovered super-enhancer heterogeneity between breast cancer subtypes. Our data also revealed TCOF1 as a putative TNBC-specific super-enhancer-regulated gene. TCOF1 plays a critical role in craniofacial development but its function in cancer remains unclear.

**Methods:**

Overall survival and multivariant Cox regression analyses were conducted using the METABRIC data set. The effect of TCOF1 knockout on TNBC growth and stemness was evaluated by in vitro and in vivo assays. RNA-seq and rescue experiments were performed to explore the underlying mechanisms.

**Results:**

TCOF1 is frequently upregulated in TNBC and its elevated expression correlates with shorter overall survival. TCOF1 depletion significantly inhibits the growth and stemness of basal-like TNBC, but not of mesenchymal-like cells, highlighting the distinct molecular dependency in different TNBC subgroups. RNA-seq uncovers several stem cell molecules regulated by TCOF1. We further demonstrate that KIT is a downstream effector of TCOF1 in mediating TNBC stemness. TCOF1 expression in TNBC is regulated by the predicted super-enhancer.

**Conclusions:**

TCOF1 depletion potently attenuates the growth and stemness of basal-like TNBC. Expression of TCOF1 may serve as a TNBC prognostic marker and a therapeutic target.

## Introduction

Breast cancer remains the second most common cause of death of women worldwide despite recent advances in diagnosis and treatment [1]. The triple-negative subtype—a form of breast cancer where tumour cells do not express oestrogen receptor and progesterone receptor and lack HER2 overexpression—is highly aggressive and with limited treatment options [2]. The heterogeneity and enrichment of cancer stem cells (CSCs) in triple-negative breast cancer (TNBC) contribute to high malignancy, resistance to therapies and frequent tumour recurrence for this subtype [3–6]. Indeed, molecular signature profiling further characterised TNBC into six categories that display unique biological characteristics, namely, basal-like 1 (BL1) and BL2, immunomodulatory (IM), mesenchymal (M), mesenchymal stem-like (MSL) and luminal androgen receptor (LAR) [7]. Understanding how CSCs are regulated in different breast cancer subgroups is therefore of key importance for future treatment strategies of TNBC.

Super-enhancers are large clusters of enhancers that drive specific expression programmes, which define cellular identity [8]. It has been demonstrated that super-enhancers also play a critical role in upregulating the expression of cancer-driver genes [9]. For example, focal amplifications of super-enhancers that drive MYC expression were identified in multiple epithelial cancers [10]. In addition, transcriptional diversity and clinical behaviours in different subgroups of medulloblastoma was exemplified by subgroup-specific super-enhancers [11]. However, an understanding of super-enhancers enriched in different subtypes of breast cancer and their functional importance are lacking. By performing network-based, epigenomic analysis, our recent work revealed that clustering of super-enhancers is sufficient to characterise breast cancer subtype identity [12]. Combining integrative network analysis and Crispr/Cas9 editing, we further identified FOXC1 as a key regulator of tumour growth and invasion driven by a TNBC-specific super-enhancer, as well as provided evidence for the functional significance of super-enhancers in determining biological outcomes. A number of super-enhancer candidate target genes were emerged in the study, highlighting the power of exploring epigenetic circuitry in TNBC for discovering novel cancer-related genes. We conducted a literature review, and among the candidate target genes, we decided to focus on TCOF1 for the following reasons: (1) TCOF1, also known as treacle, has been demonstrated to play key roles in neural crest formation and ribosome biogenesis [13–16]. Haploinsufficiency of TCOF1 results in Treacher Collins syndrome (TCS), one of the most severe congenital disorder of craniofacial development [17], highlighting its importance in cell proliferation and growth at the developmental stage. Recently, TCOF1 has been implicated in DNA damage response via interaction with NBS1 and MRNM in neuroepithelium [18, 19]. (2) Disease associated with overexpression of TCOF1, however, has not been described. (3) The role of TCOF1 in the pathogenesis of cancer is yet to be identified. (4) Our preliminary data indicated that TCOF1 is highly expressed in TNBC cell lines but not in luminal lines, and the lack of TCOF1 expression in normal breast tissues suggests the potential for therapeutic development.

In the present study, we conducted functional analyses of TCOF1 in vitro and in vivo and identified its critical oncogenic function in basal-like subtype of TNBC. We uncovered the clinical significance and crucial role of TCOF1 in regulating TNBC growth and stemness, as well as the regulation of TCOF1 expression by a TNBC-specific super-enhancer. Our findings further determined KIT as a downstream molecule of TCOF1 in mediating its function in CSC self-renewal.

## Materials and methods

### Cell culture

MDA-MB-231, MDA-MB-436, MDA-MB-468, Hs578T, ZR-75-1, HCC38, HCC1143, HCC70, HCC1806, BT-549, T47D, BT474, MCF-7 and HEK293T cells were obtained from ATCC. MCF10-DCIS and SUM159-PT cells were provided by Kornelia Polyak (Harvard Medical School, USA). Cells were maintained as described in [12]. Briefly, MDA-MB-468, MDA-MB-231, T47D, MCF-7 and HEK293T cells were cultured in Dulbecco’s Modified Eagle medium (DMEM; Gibco) containing 10% Tet system-approved foetal bovine serum (FBS; Clontech). HCC70, HCC1806, ZR-75-1, HCC38, HCC1143 and BT-549 cells were maintained in RPMI 1640 medium (Gibco) containing 10% FBS. Hs578T and MDA-MB-436 were maintained in DMEM supplemented with 10% FBS and 10 µg/ml insulin. MCF10-DCIS [20] was maintained in DMEM/F-12 (Gibco) supplemented with 5% horse serum, 20 ng/ml epidermal growth factor (EGF), 10 µg/ml insulin, 100 ng/ml final cholera toxin and 500 ng/ml hydrocortisone. SUM159-PT was maintained in Ham’s F12 Medium (Lonza) supplemented with 5% FBS, 5 µg/ml insulin and 500 ng/ml hydrocortisone. BT474 was maintained in RPMI 1640 medium supplemented with 10% FBS and 10 µg/ml insulin. All cell lines are routinely assayed for mycoplasma contamination. They have been tested for authentication using short tandem repeat profiling and passaged for <6 months.

### Antibodies

Anti-KIT (#3074S), anti-HA tag (#3724S), anti-phospho-KIT(T719) (#3391), anti-phospho-ERK (#4370T), anti-phospho-STAT3(T705) (#9145T), anti-phospho-AKT(T473) (#9271) and anti-actin (#3700) antibodies were obtained from Cell Signaling Technology. Anti-TCOF1 (#HPA038237) and anti-FZD8 (#HPA045025) antibodies were purchased from Sigma-Aldrich. Horseradish peroxidase-conjugated anti-mouse and anti-rabbit immunoglobulin G (IgG) antibodies (AP307P, AP308P) were purchased from Millipore. Anti-Brd4 (Bethyl Laboratories, #A301-985a100) and anti-H3K27ac (Active motif, #39685) were used for chromatin immunoprecipitation–quantitative polymerase chain reaction (ChIP-qPCR). Anti-ribosomal RNA antibody (Y10B) was purchased from Abcam (#ab171119).

### Plasmids

To knock out TCOF1 and KIT, Crispr/Cas9 knockout system was used. FUCas9Cherry (#70182), lentiV_Cas9_puro (#108100) and FgH1tUTG (#70183) were ordered from Addgene. Guide RNAs (gRNAs) was designed using online tool www.crisprscan.org/ and http://crispr.mit.edu/. gRNA oligos (Table S1) with sticky end were synthesised by IDT company. Then gRNAs were cloned into BsmBI restriction site of FgH1tUTG vector. To overexpress endogenous TCOF1, CRISPR/Cas9 Synergistic Activation Mediator (SAM) system was used; vectors of lenti-dcas-vp64_blast (#61425), lenti-sgRNA(MS2)_puro (#73795) and lentiMPH v2 (89308) were purchased from Addgene. To overexpress exogenous HA-TCOF1, CDS of TCOF1 with HA tag at N-terminal was synthesised and cloned into CD532A-1 vector by GENEWIZ. To overexpress exogenous FZD8-HA, KIT-HA, CDS of FZD8 and KIT with HA tag at C-terminal were synthesised and cloned into CD532A-1 vector by GENEWIZ. To delete super-enhancer peak, http://crispr.mit.edu/ was used to design a pair of gRNA franking the peak. The gRNA pairs were then inserted into BbsI and BsaI restriction sites of px333 vector (Addgene 64073). PCR was employed to amplify hU6 promoter-sgRNA-hU6 promoter-sgRNA. The sequence was then cloned into the PacI-digested lentiviral vector FgH1tUTG.

### Lentivirus infection

To produce lentiviral supernatants, 10 µg lentiviral vectors (FgH1tUTG, FUCas9Cherry, lentiV_Cas9_puro, CD532A-1, lenti-dcas-vp64_blast, lenti-sgRNA(MS2)_puro or lentiMPH v2) were co-transfected with 7 µg psPAX2 and 2.4 µg VSV-G vectors to HEK293T cells, using polyethylenimine as transfection reagent. Sixty hours post transfection, lentiviruses were filtered by 0.45 µm syringe filter (Thermo fisher 7232545). In all, 0.3–0.75 ml lentivirus with 5 µg/ml polybrene were added to breast tumour cells for 12–24 h in a well of 6-well plates. Cells were sorted by fluorescence-activated cell sorting (FACS) with a cell sorter (Sony) or selected with puromycin, blasticidin or hygromycin for 5–7 days.

### Identification of TNBC-specific super-enhancer target–gene pairs

ChIP-seq and gene expression analyses were performed as described in [12]. Briefly, ChIP-seq data for H3K27ac (8 cell lines) and DNase-seq data of MDA-MD-231 were downloaded from Gene Expression Omnibus (GEO; https://www.ncbi.nlm.nih.gov/geo/) and EMBL-EBI ArrayExpress (https://www.ebi.ac.uk/). H3K27ac ChIP-seq reads were mapped to the human reference genome (UCSC hg19 genome build) using Bowtie (version 1.2.2) [21], and uniquely mapped reads were retained for downstream analyses. Coverage tracks were generated for each mark with ‘deeptools’ (version 3.3.0) and histone modification enrichment signals were scaled by Counts Per Million mapped reads [22]. For each cell line, a two-state hidden Markov model (ChromHMM, version 1.17) [23] was used to find H3K27ac enrichment regions. To retain regions as predicted enhancers, H3K27ac enrichment regions located at gene promoters (within +/−2.5 kb of transcriptional start site) were filtered out. Based on LOESS regression by fitting the enhancer size distribution and the inflection point (slope 1), super-enhancers were distinguished from typical enhancers. DNase peaks (*p* < 1 × 10^−5^) for MDA-MB-231 were identified using the MACS software (version 2.1.0) [24]. Two DNase peaks were identified in the super-enhancer of TCOF1 and the major peak e1 was further employed for luciferase reporter assays and Crispr/Cas9 studies.

The use of TCGA level-3 gene expression data of 605 non-TNBC and 115 TNBC tissue samples (termed ‘TCGA-BRCA’ data set) in gene expression quantification is described in [12]. Briefly, ‘limma’ (R package, version 3.32.2) [25] was used to identify genes that are significantly upregulated (log2 fold change >0.5, Benjamini–Hochberg-adjusted *p* < 0.05) in TNBC samples, compared to non-TNBC samples. A total of 331 TNBC-specific super-enhancer target–gene pairs involved in cancer hallmarks were obtained.

### Immunoblotting

Cells were lysed in EBC buffer (0.5% NP-40, 120 mM NaCl, 50 mM Tris-HCl (pH 7.4), proteinase inhibitor cocktail, 50 nM calyculin, 2 mM EDTA, 2 mM EGTA, 1 mM sodium pyrophosphate, 20 mM sodium fluoride) on ice for 25 min. Cell extracts were pre-cleared by centrifugation at 13,000 × *g* at 4 °C for 10 min and protein concentration was measured with the Bio-Rad protein assay reagent using a BioTek Synergy™ H1 Microplate Reader. Lysates were then resolved by sodium dodecyl sulfate-polyacrylamide gel electrophoresis and detected with the indicated antibodies and enhanced chemiluminescence substrate (Pierce).

### Immunofluorescence

Cells were fixed with 4% paraformaldehyde (PFA) in phosphate-buffered saline (PBS) for 20 min at room temperature. Cells were then permeabilised by incubating with 0.5% TritonX-100/PBS for 2 min. Glycine buffer (0.75% glycine in PBS) was used to rinse cells for 3 times. Cells were then incubated with blocking buffer (10% goat serum, 0.2% TritonX-100 in PBS) for 45 min followed by incubation with primary antibody (anti-TCOF1, 1:200 or anti-rRNA, 1:200) for 2 h and 1.5 µg/ml Alexa Fluor 647 Goat anti-Rabbit secondary antibody (Jackson ImmunoResearch #111-605-003) for 1 h. The LSM880 (Zeiss) laser scanning microscope or Thermo Scientific CellInsight CX7 High-Content Screening (HCS) Platform was used to capture images.

### Immunohistochemistry (IHC) of clinical breast cancer samples

IHC was performed as described in [12]. Briefly, we acquired breast tissue samples from breast cancer patients who underwent biopsy or surgery for resection. Formalin-fixed paraffin-embedded breast tissue sections (5 µm) were prepared. Sections were pre-heated at 90–95 °C in Envision Flex Target Retrieval Solution of low pH (DAKO, Denmark, K8005) to retrieve antigens and microwaved for further 15 min. Sections were cooled down in the retrieval solution for 20 min at room temperature, followed by incubation with rabbit anti-TCOF1 antibody (Sigma, HPA038237, dilution 1:100) for 30 min at room temperature. REAL EnVision Detection System and DAB chromogenic substrate (DAKO, Denmark, K5007) were then incubated with sections for 30 and 2 min, respectively, at room temperature for signal detection. Haematoxylin was used for nuclei counterstain. Stained sections were semi-quantitatively scored according to their staining intensity with grade ≤2.0 as negative/weak staining (low TCOF1), grade 2.0–3.5 as moderately strong staining (moderate TCOF1) and grade ≥3.5 as the strongest staining (high TCOF1). The procedures were approved by the Human Subjects Ethics Committees at City University of Hong Kong and conformed to the government regulations for research involving human participants. Informed consent was obtained from the breast cancer patients.

### Survival analyses and clinicopathologic features in clinical cohorts of breast cancer

The association of TCOF1 mRNA expression with patient survival was assessed using the Molecular Taxonomy of Breast Cancer International Consortium (METABRIC) discovery data set [26], including a total of 1953 breast cancer cases with clinical follow-up. We plotted Kaplan–Meier curves in TCOF1-high expressing versus TCOF1-low expressing tumours. ‘Maxstat’ R package was used to compute the maximally selected log-rank statistic for cutpoints, in order to define the cutpoint that provides the best separation into two groups. The significance in difference between survival curves was evaluated by log-rank tests. Clinical variables included age at diagnosis, tumour grade (1, 2, 3), Tumour, Node, Metastasis (TNM) stages (I, II, III, IV) and tumour size (≤5 cm and >5 cm). *χ*^2^ test, Fisher exact test or *t* test were used to assess the differences in distribution of clinical variables in TCOF1-low expressing and TCOF1-high expressing groups. To determine whether TCOF1-high expression is an independent prognostic factor, multivariate Cox regression model was used.

### Clonogenic growth assays

Cells were seeded to 6-well plate and cultured for 2 weeks. Medium was changed every 4 days. After 2 weeks, cells were fixed with 4% formaldehyde for 15 min in room temperature. Colonies were stained with 0.1% crystal violet for 40 min followed by PBS wash. To count the number of colonies, pictures were captured, and colony number was counted using the ImageJ software. Cell proliferation was quantified by destaining cells in 10% acetic acid, followed by reading the optical density value at 595 nm on a spectrophotometer [10].

### CellTiter-Glo® three-dimensional (3D) and two-dimensional (2D) cell viability assays

3D cultures were prepared as previously described [27]. Briefly, growth factor-reduced Matrigel (BD Biosciences) was used to coat 96-well plates (Corning #3610). In all, 1500–3000 cells were seeded in Assay medium, containing DMEM or RPMI 1640 supplemented with 10% FBS and 2% Matrigel. To quantify spheroid growth, CellTiter-Glo® 3D Cell Viability Assay (Promega #G9682) was carried out followed the instruction of the product manual. For 2D culture, 3000 cells per well were seeded to 96-well plate and cultured for 3 days. CellTiter-Glo® Luminescent Cell Viability Assay (Promega #G7571) was used to quantify cell viability.

### Mammosphere-formation assay

For first generation of mammosphere formation, cells were seeded to ultra-low attachment 6-well plate (Corning 3471) with cell density of 1000 and 2500 cells per well for HCC1806 and MDA-MB-468 cells, respectively. Cells were cultured in mammosphere medium, containing DMEM/F12 supplemented with B27 (Gibco 12587010) and 20 ng/ml EGF (R&D 236-EG) for 5–6 days. Images of mammospheres were captured by the Nikon Eclipse Tis2 microscope at ×4 magnification objective. Number of mammospheres with diameter ≥70 µm were counted using the Nikon NIS-Elements D software. Second-generation mammosphere-formation assay was performed as previously described [28]. Briefly, mammospheres from first generation were collected, dissociated by trypsin and washed by mammosphere medium once. All cells were then seeded to a new ultra-low attachment 6-well plate with similar densities and cultured for 5–6 days. Ratio of second-generation mammosphere number to first-generation mammosphere number was calculated.

### AldeRed aldehyde dehydrogenase (ALDH) detection assay

AldeRed ALDH Detection Assay Kit (Merck, #SCR150) was used to detect ALDH activity of cells according to the manufacturer’s instruction. Briefly, cells in 2D culture were treated with 100 ng/ml doxycycline (dox) for 6 days to induce TCOF1 knockout (here LentiV_cas9_puro was used rather than FUCas9cherry), followed by culturing in 3D for 5–6 days. Spheroids were collected and dissociated by trypsin. In all, 2 × 10^5^ cells were then incubated with AldeRed reagent and verapamil for 35 min in 37 °C, protected from light. Cells were centrifuged and pellets were resuspended with 0.5 ml ice-cold ALDE-Red buffer on ice. ALDE-Red signal was detected by Beckman Coulter CytoFLEX S Flow cytometer analyser, using ECM detector (610/20 BP). DEAB was used as a negative control to establish baseline fluorescence.

### Reverse transcription (RT)–qPCR

Mammosphere samples were cultured with mammosphere medium in ultra-low attachment 10-cm plates for 6 days. 3D spheroids were cultured in ultra-low attachment 6-well plates with PRMI-1640 or DMEM supplemented with 10% Tetracycline Free FBS and 2% Matrigel for 5 days. Total RNA from 3D culture and mammosphere culture were extracted using the RNeasy Plus Mini Kit (Qiagen #74134) following the manufacturer’s instruction. RT was performed using TaqMan Reverse Transcription Reagents (Applied Biosystems, N8080234). Quantitative RT-PCR was performed using a QuantStudio 12K Flex Real-Time PCR System (Applied Biosystems).

### RNA-seq analysis of mammospheres

MDA-MB-468 cells with or without TCOF1 knockout were seeded to 10-cm ultra-low attachment plates (6–7 plates per group) and cultured with mammosphere culture medium for 7 days. Mammosphere were collected and total RNA was extracted using TRIzol reagent (Invitrogen, CA, USA) according to the manufacturer’s manual. The RNA library construction and RNA-seq analysis was performed by the HaploX Genomics Center (Shenzhen, China). Experiment was performed without biological replicates. For RNA-seq analysis, we used the fastp software [29] to trim adaptor and used the FastQC software to perform quality-control assessment of fastq file. Gene expression raw counts were computed by STAR mapping with gene quantification mode. GFOLD V1.1.4 with default parameters was used to analyse the differential gene expression, which provided reliable log fold change based on posterior distribution [30]. HTSanalyzeR2 R package [31] was used to perform gene set enrichment analysis (GSEA) based on log fold change given by GFOLD.

### ChIP-qPCR

ChIP-qPCR was performed as described in [12]. Briefly, cells were incubated with 1% PFA at room temperature for 5 min for crosslinking. After washing twice with PBS, cells were harvested by scraping in 1 ml ChIP lysis buffer (1% Triton, 0.1% Na-deoxycholate, 50 mM HEPES, pH7.5, 140 mM NaCl, 1 mM EDTA, 1× proteinase inhibitor cocktail) and incubated on ice for 15 min. Cells were sonicated using Bioruptor Plus (Diagenode, UCD-300 TM) for 30 cycles (30 s ON and 30 s OFF at high power) to shear the chromatin, followed by two sequential high-speed centrifugations (10,000 × *g* for 5 and 15 min at 4 °C) to collect the soluble chromatin. Lysate was incubated with the indicated antibodies at 4 °C for overnight, followed by incubating with pre-washed Protein G Sepharose (GE Healthcare, 17061802) at 4 °C for 1 h. After washing, samples were incubated with 10% chelex (Bio-Rad, cat. no. 142–1253) and then with 20 mg/ml Proteinase K (NEB, P8107S), followed by centrifugation. qPCR was performed by Applied Biosystems QuantStudio 3 Real-Time PCR System.

### Dual luciferase reporter assay

Fragments containing enhancer e1 of TCOF1-associated super-enhancer was amplified from genomic DNA by PCR. Using MluI and XhoI restriction enzyme, e1 enhancer was cloned upstream of the promoter-luc+ transcriptional unit of firefly luciferase reporter pGL3-Promoter vector (Promega, #E1761). FuGENE 6 (Promega, #2693) was then used to co-transfect the enhancer luciferase constructs with pRL-TK vector (Promega, #E2241) into BT-549 cells. We used the renilla luciferase reporter pRL-TK vector as an internal control reporter vector. Two days post-transfection, luminescence signal was quantified using the Dual-Glo® Luciferase Assay System Kit (Promega, #E2920). The firefly luciferase signal was first normalised to the renilla luciferase signal and then normalised to the empty pGL3-promoter plasmid signal.

### PCR for verification of super-enhancer deletion

To confirm the Crispr/cas9-mediated deletion of super-enhancer peak, genomic DNA was extracted using the QIAamp DNA Mini Kit (Qiagen #51304), followed by PCR to amplify target sequences with or without deletion. Quick-Load® Taq 2× Master Mix (M0270L) was purchased from NEB. Primers flanking the deleted region are designed using Primer-blast online tool and synthesised by Integrated DNA Technologies company (Table S1). PCR products were resolved on a 1.3% agarose gel.

### Prediction of potential transcription factors (TFs) at super-enhancer

To identify binding TFs, the DNase peak e1 of the TCOF1 super-enhancer region was used [12]. The potential binding sites for TFs were detected at the nucleosome-free regions using FIMO [32] with default parameters (*p* < 1 × 10^−4^), together with position frequency matrices from JASPAR database (http://jaspar.genereg.net/) [33].

### Xenograft studies

Six–8-week-old female nude mice were obtained from the laboratory animal services centre, Chinese University of Hong Kong. All mice were in good health status. All procedures were approved by the Animal Ethics Committees at City University of Hong Kong and conform to the government guidelines for the care and maintenance of laboratory animals. Randomisation was used to allocate experimental units to control and treatment groups. Ear tags with unique number were used for labelling mice. Investigator was blinded to the group allocation during the experiment. To assess tumour growth in vivo, cells cultured in 2D were treated with or without 100 ng/ml dox for 6 days. In all, 4 × 10^6^ cells in 0.1 ml media with 50% Matrigel were injected into mammary fat pads (MFPs) of the mice using 1-ml syringe. Tumour formation was examined every 2–3 days. Tumour volumes were calculated using the formula *V* = (*W*^2^ × *L*)/2, where *V* is the tumour volume, *W* is the tumour width and *L* is the tumour length. Tumour weight was measured by analytical balance. For in vivo limiting dilution assays, cells pre-treated with or without 100 ng/ml dox were seeded and cultured in 3D for 5 days. Then spheroids were collected, dissociated by trypsin and resuspended in 50% Matrigel/PBS. For HCC1806, 300, 1200 and 4800 cells were implanted into the fourth MFP of nude mice. For MDA-MB-468, 400, 2000 and 10,000 cells were implanted. Online tool ELDA (http://bioinf.wehi.edu.au/software/elda/) was used to calculate tumour-initiating cell frequency and *p* value.

### Statistical analysis

Statistical significance between conditions was assessed by Student’s *t* tests. In all the figures, data are presented as mean ± standard error of the mean (SEM). Significance between conditions is denoted as *p < 0.05, **p < 0.01 and ***p < 0.001. At least three independent experiments were performed for each condition for verification of the emphasised trends in in vitro studies. For xenograft studies, at least six tumours in each condition were analysed.

## Results

### TCOF1 is highly expressed and correlated with poor overall survival in TNBC patients

**Fig. 1:**
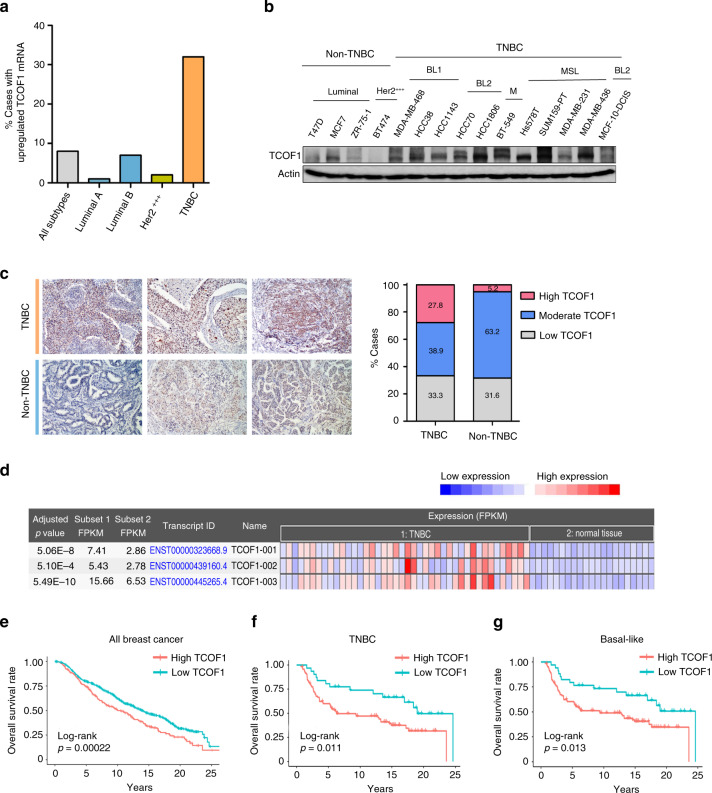
Upregulation of TCOF1 in TNBC is correlated with poor prognosis. **a** Bar graph depicting the percentage of breast cancer cases with upregulated TCOF1 mRNA expression, using a data set from TCGA (TCGA, Cell 2015 data set). mRNA expression *z*-score was set to ±1.5 as a threshold. *z*-score >1.5 is considered as upregulated expression. **b** Immunoblotting showing expression of TCOF1 in a panel of breast cancer cell lines. Her2 +++ Her2-overexpressed, BL1 basal-like, BL2 basal-like 2, M mesenchymal, MSL mesenchymal stem-like. Experiments in **b** were repeated twice independently with similar results. **c** Left panel showing representative IHC pictures of TNBC and non-TNBC. Graph showing the percentage of breast cancer cases displaying low, moderate, and high staining intensity of TCOF1 by IHC. Number of cases for TNBC and non-TNBC are 18 and 19, respectively. **d** TCOF1 mRNA expression in TNBC (Subset 1; *n* = 42) and normal adjacent tissue to TNBC (Subset 2; *n* = 21), data from Cancer RNA-seq Nexus. Expression data of three major transcripts are shown. **e** Kaplan–Meier survival analysis of breast cancer patients with high (number of patients *n* = 224) and low (number of patients *n* = 735) TCOF1 expression. The optimal cutpoint = 5.51. **f** Kaplan–Meier survival analysis of TNBC patients with high (number of patients *n* = 94) and low (number of patients *n* = 31) TCOF1 expression. Normalised TCOF1 mRNA values of all breast cancer cases have a normal distribution, with a median of 5.39. The optimal cutpoint = 5.35. **g** Kaplan–Meier survival analysis of basal-like breast cancer patients with high (number of patients *n* = 105) and low (number of patients *n* = 34) TCOF1 expression. The optimal cutpoint = 5.35. The *p* value in **e**–**g** was calculated by log-rank test (one-sided).

In this study, we aimed at leveraging our multiomic analysis of super-enhancer landscape to identify novel players in TNBC. With TNBC-specific super-enhancer profiling and gene expression analysis using TCGA-BRCA data set, a total of 331 TNBC-specific super-enhancer target–gene pairs involved in cancer hallmarks were obtained (for details of analysis, see [12] and ‘Materials and methods’). As there is no report on the role of TCOF1 in cancer, nor any association of TCOF1 overexpression with any disease, here we focussed on the SE324–TCOF1 pair and determined the functional importance of TCOF1 in breast tumorigenesis. Super-enhancer SE324 is mapped approximately 38 kb upstream of TCOF1 [12]. To investigate whether TCOF1 is overexpressed in TNBC, we analysed a data set of breast cancer from The Cancer Genome Atlas Project (TCGA Cell 2015). Whereas TCOF1 mRNA is upregulated in 32% TNBCs, its expression is underrepresented in other subtypes of breast cancer (Fig. 1a). In addition, in a panel of breast tumour lines, protein expression levels of TCOF1 are higher in TNBC lines as compared to luminal or HER2-overexpressed lines (Fig. 1b). These observations were further validated by IHC in a cohort consisting of 37 primary breast cancer cases, in which higher percentage of TNBC patients had high staining intensity of TCOF1 compared to non-TNBC cases (Fig. 1c). Importantly, TCOF1 is minimally expressed in normal breast tissues adjacent to TNBC (Cancer RNA-seq Nexus database (Fig. 1d). To evaluate the association of TCOF1 mRNA expression with patient overall survival, we conducted Kaplan–Meier survival analysis on the METABRIC data set. In all patients, those with higher TCOF1 expression had significantly shorter overall survival than those with lower TCOF1 expression (*p* = 0.00022; Fig. 1e). In particular, high TCOF1 expression in TNBC or basal-like breast cancer resulted in a significant shorter survival than those with low TCOF1 expression (*p* = 0.011 and 0.013, respectively; Fig. 1f, g). Furthermore, by interrogating clinical data sets containing 959 breast cancer cases, we found that high expression of TCOF1 was significantly associated with tumour grade (*p* < 0.001) and TNM stage (*p* < 0.001) (Table S2), suggesting that TCOF1 may enhance tumour growth in TNBC patients. High expression of TCOF1mRNA was also found to be an independent risk factor for shortened survival in TNBC patients by multivariant Cox regression analysis (relative risk, 2.71; 95% confidence interval,1.31–5.6; Table S3).

### Knockout of TCOF1 inhibits basal-like TNBC spheroid growth and tumour growth in vivo

**Fig. 2:**
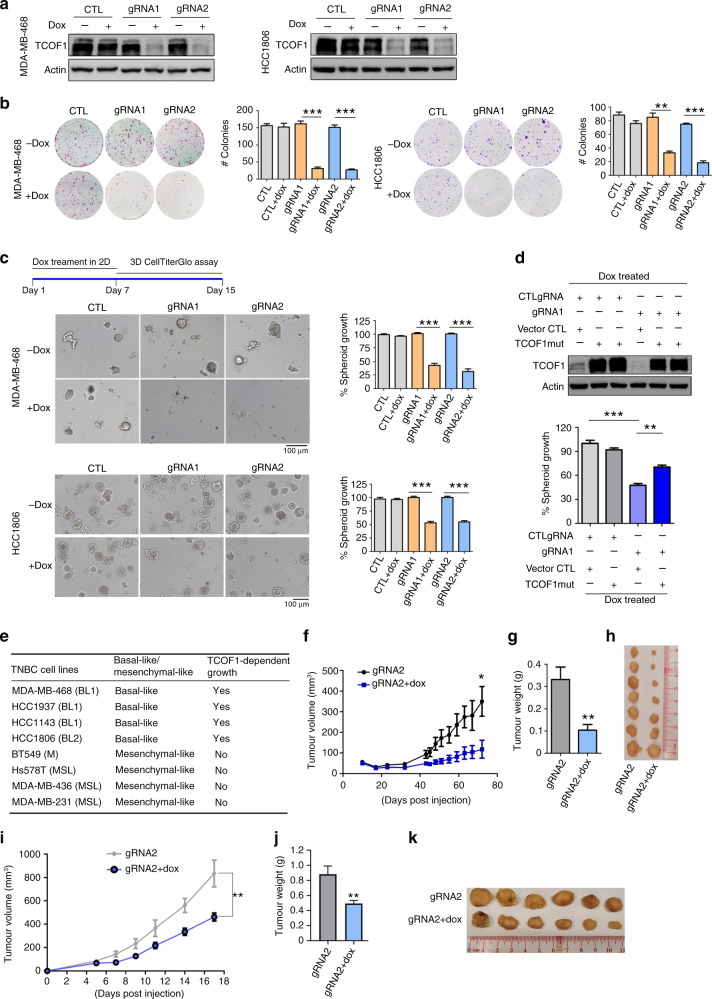
TCOF1 promotes TNBC spheroid growth and tumour growth in vivo. **a** MDA-MB-468 and HCC1806 cells expressing tet-on TCOF1 gRNA or vector control (CTL) were treated with doxycycline (dox; 100 ng/ml) for 5 days. Whole-cell lysates were subjected to immunoblotting. Experiments in **a** were repeated >3 times independently with similar results. **b** MDA-MB-468 and HCC1806 cells with or without TCOF1 knockout were cultured for colony-formation assay for 14 days. Representative images are shown. Colony number was counted and depicted in the bar graph. Error bars, mean ± SEM of three independent experiments. **p < 0.01; ***p < 0.001. **c** Schematic of 3D CellTiter-Glo assay. Left panel, representative pictures of spheroids. Bar graphs depict growth of MDA-MB-468 and HCC1806 spheroids with or without TCOF1 knockout. Error bars, mean ± SEM of three independent experiments. ***p < 0.001. **d** MDA-MB-468 cells expressing TCOF1 with a mutation on PAM sequence (TCOF1 mut) or control vector were infected with tet-on TCOF1 or CTL gRNA. Cells were cultured in 3D for 6 days, followed by 3D CellTiter-Glo assay. Error bars, mean ± SEM of three independent experiments. **p < 0.01; ***p < 0.001. Whole-cell lysates were subjected to immunoblotting. **e** Table summarising the effect of TCOF1 knockout on spheroid growth of different TNBC lines. **f**, **g** MDA-MB-468 xenograft growth (**f**) and tumour weight (**g**) upon TCOF1 knockout. Error bars, mean ± SEM (number of tumour of each condition *n* = 7). *p < 0.05; **p < 0.01. **h** Picture of MDA-MB-468 tumours**. i**, **j** HCC1806 xenograft growth (**i**) and tumour weight (**j**) upon TCOF1 knockout. Error bars, mean ± SEM (number of tumour of each condition *n* = 6). **p < 0.01. **k** Picture of HCC1806 tumours. *p* values were calculated by two-sided Student’s *t* test in **b**–**d**, **f**, **g**, **i**, **j**.

To functionally determine the pathological role of TCOF1 in TNBC, we constructed a panel of TNBC lines with basal-like morphology using tet-on dox-inducible, Crispr/Cas9-mediated knockout of TCOF1. After lentiviral infection, double-positive cells with both mCherry (spCas9) and green fluorescent protein (dox-inducible sgRNAs) were sorted by FACS (Fig. S1A). Upon dox administration, TCOF1 was depleted significantly with two distinct gRNAs (Figs. 2a and S1B, C). Crispr/Cas9-mediated DNA editing on TCOF1 gene were validated by sequencing (Fig. S1D). We then investigated the consequence of TCOF1 knockout on progeny-producing capability of TNBC cells. TCOF1 knockout potently inhibited colony formation in clonogenic assays (Fig. 2b). Furthermore, depletion of TCOF1 in TNBC lines MDA-MB-468 and HCC1806 inhibited spheroid growth significantly in 3D cultures (Fig. 2c), which more accurately recapitulates phenotypes that govern tumour growth in vivo. TCOF1 knockout also attenuated TNBC cell proliferation in 2D culture (Fig. S2A, B). Importantly, overexpression of TCOF1 with a silent point mutation in PAM sequence could partially rescue spheroid growth of TCOF1 knockout cells (Fig. 2d). We next addressed the potential combinatorial effects of TCOF1 depletion and chemotherapeutic agents on TNBC growth. Whereas treatment of cells with cisplatin or TCOF1 knockout alone led to ~50% reduction of spheroid growth, combination treatment resulted in 75% growth inhibition (Fig. S2C, left). Similar results were observed when TCOF1-knockout cells were treated with paclitaxel **(**Fig. S2C, middle). TCOF1 has been shown to be phosphorylated by CK2 kinase during embryogenesis [34], and CK2 inhibitor suppresses viability of TNBC CSCs [35]. The combinatorial effects of TCOF1 knockout and CK2 inhibitor Silmitasertib were therefore being examined in our 3D TNBC model. We showed that combined treatment resulted in 80% of inhibition on spheroid growth (Fig. S2C, right). To further assess the role of TCOF1 in different subgroups of TNBC, we performed knockout studies in a diverse set of TNBC lines. In addition to BL1 line (MDA-MB-468) and BL2 line (HCC1806), we analysed two additional BL1 lines (HCC1937, HCC1143), one M line (BT549) and 3 MSL lines (Hs578T, MDA-MB-436, MDA-MB-231). Interestingly, whereas TCOF1 depletion had potent effect on spheroid growth in basal-like TNBC lines, it had no effect on mesenchymal-like spheroids (Figs. S2D–I and 2e). In the TCS model, loss of TCOF1 function resulted in the downregulation of rDNA transcription [13], we therefore quantified levels of 45S and 5S ribosomal RNAs (rRNAs), as well as performed immunofluorescence to exam the integrity of rRNA in TNBC cells. Our data showed that TCOF1 knockout did not lead to reduction of 45S or 5S rRNA (Fig. S3A), nor impair rRNA integrity (Fig. S3B) in TNBC cells. We also assessed the effect of TCOF1 on cell death and demonstrated that TCOF1 depletion did not induce TNBC cell death in 3D or 2D cultures (Fig. S3C, D). We next extended our studies in vivo, in which TNBC cells with TCOF1 knockout were injected orthotopically into nude mice. Depletion of TCOF1 in basal-like MDA-MB-468 cells resulted in a profound decrease of tumour growth (Fig. 2f–h). TCOF1 knockout in another basal-like TNBC line HCC1806 had similar effect on the inhibition of xenograft growth (Fig. 2i–k).

### TCOF1 depletion attenuates stemness of TNBC

**Fig. 3:**
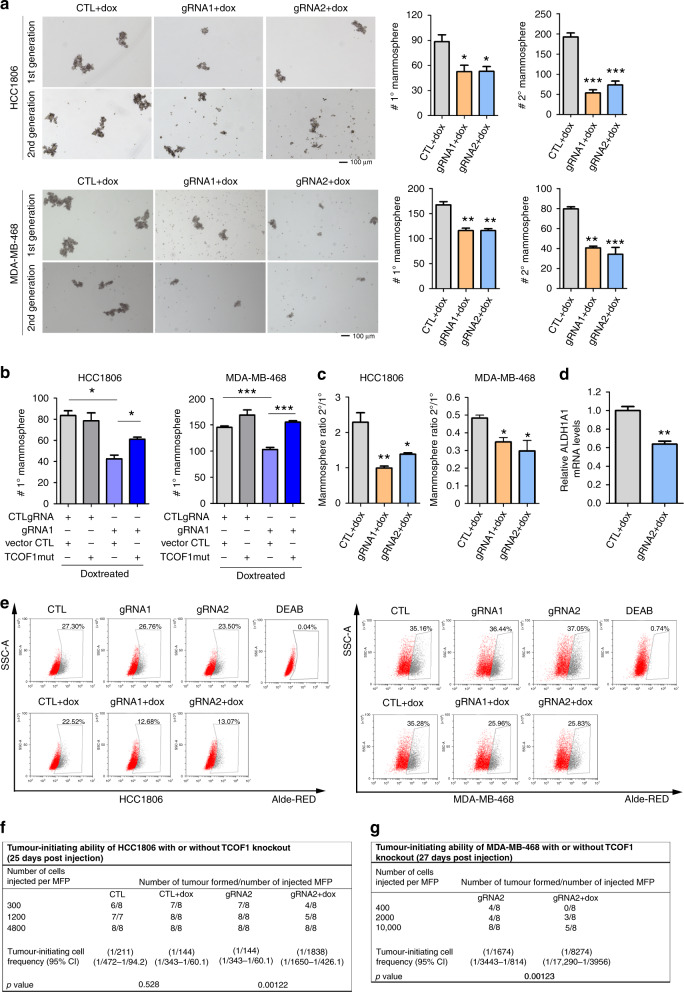
TCOF1 depletion attenuates stemness of TNBC CSCs. **a** MDA-MB-468 and HCC1806 cells expressing tet-on TCOF1 or CTL gRNA were treated with dox (100 ng/ml) for 5 days. Cells were then seeded for first- and second-generation mammosphere-formation assay. Left panel, representative pictures of mammosphere. Right panel, bar graphs depict the mammosphere number. Error bars, mean ± SEM of three independent experiments. *p < 0.05; **p < 0.01; ***p < 0.001. **b** MDA-MB-468 cells expressing TCOF1 mut or control vector were infected with tet-on TCOF1 or CTL gRNA. Cells were treated with dox (100 ng/ml) for 5 days and then subjected to mammosphere-formation assay. Error bars, mean ± SEM of three independent experiments. *p < 0.05; ***p < 0.001. **c** Ratio of secondary to primary mammosphere number of HCC1806 and MDA-MB-468 lines. Error bars, mean ± SEM of three independent experiments. *p < 0.05; **p < 0.01. **d** mRNA level of ALDH1A1 in MDA-MB-468 with or without TCOF1 knockout. Error bars, mean ± SEM of three independent experiments. **p < 0.01. **e** ALDH activity of cells derived from HCC1806 and MDA-MB-468 spheroids with or without TCOF1 knockout, measured by AldeRed ALDH detection assay. **f**, **g** Limiting dilution analysis of tumour-initiating cell frequency of HCC1806 and MDA-MB-468 cells with or without TCOF1 knockout. Tumour incidence was shown as number of tumour formed/number of injected MFP. Tumour-initiating cell frequency was calculated using the ELDA software. Cl confidence interval. *p* values were calculated by two-sided Student’s *t* test in **a**–**d**.

Since our data of clonogenic assay suggest that TCOF1 regulates progeny producing capability, we next investigated if TCOF1 regulates CSC properties and tumour-initiating ability. In mammosphere-formation assays, we found that both first and second generation of mammosphere number reduced significantly upon TCOF1 knockout (Fig. 3a). Conversely, overexpression of endogenous TCOF1 by CRISPR/Cas9 SAM or exogenous HA-TCOF1 resulted in an opposing trend (Fig. S4A, B). Importantly, overexpression of TCOF1 could rescue mammosphere-formation ability of MDA-MB-468 and HCC1806 cells with TCOF1 knockout (Fig. 3b), demonstrating that TCOF1 promotes TNBC CSC properties. Self-renewal ability of CSCs can be assessed by the ratio of second- to first-generation mammosphere numbers [28]. Depletion of TCOF1 led to a reduction of the ratio (Fig. 3c), indicating that TCOF1 plays an important role in enhancing self-renewal of CSCs. We further tested the effect of TCOF1 on ALDH expression and activity, a marker of breast CSC [36]. The expression level of ALDH1A1 was reduced by 37% upon TCOF1 knockout (Fig. 3d). Knockout of TCOF1 markedly decreased the percentage of ALDH^high^ population in TNBC spheroids (Fig. 3e). Similar effects were observed in cells cultured in 2D (Fig. S4C). In addition, the percentage of CD44^high^/CD24^-/low^ cells, which have been shown to be enriched in CSCs of some TNBC lines and human breast tumours [37, 38], was decreased in TCOF1-depleted HCC1806 cells (Fig. S4D). Agreeing with previous studies [39], MDA-MB-468 cells are lack of CD44^high^/CD24^-/low^ cells (data not shown), exemplifying the significant diversity of the abundance of CD44^high^/CD24^−/low^ cells within the TNBC subtype. Interestingly, whereas TCOF1 had no effect on cell death when TNBC cells were grown in 3D or 2D (Fig. S3C, D), depletion of TCOF1 in mammosphere significantly induced cell death (Fig. S4E), suggesting a preferential regulation of CSC survival by TCOF1. To assess the degree to which TCOF1 regulates tumour-initiating ability, TNBC cells derived from spheroids with or without TCOF1 knockout were injected into MFPs of female nude mice at limiting dilutions. Mice injected with TCOF1 knockout cells showed decreased tumour incidence and significant lower frequency of tumour-initiating cells compared with other subgroups (Fig. 3f, g). There was no significant difference between dox-treated and dox-untreated control groups. Taken together, these data demonstrate an important function of TCOF1 in regulating CSC self-renewal and tumour initiation.

### KIT is a downstream effector of TCOF1 in regulating TNBC stemness

**Fig. 4:**
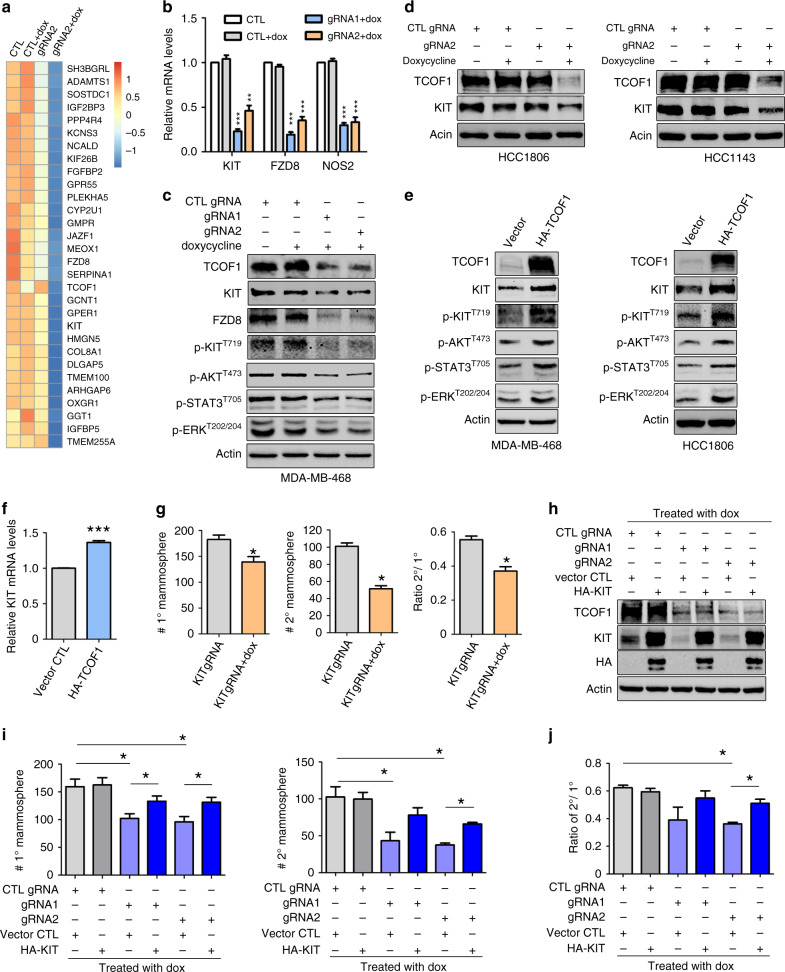
KIT is a critical downstream effector of TCOF1 in regulating CSC stemness. **a** Heatmap of downregulated genes in TCOF1-depleted MDA-MB-468 mammospheres. Cutoff, Log2 FC < −1, GFOLD < −0.5. **b** RT-qPCR verified downregulation of KIT, FZD8 and NOS2 mRNAs in MDA-MB-468 mammospheres with TCOF1 knockout. Error bars, Mean ± SEM of three independent experiments. **p < 0.01; ***p < 0.001. **c** Immunoblotting showing expression levels of the indicated proteins in MDA-MB468 spheroids with or without TCOF1 knockout. **d** Expression levels of KIT in HCC1806 and HCC1143 spheroids with or without TCOF1 knockout. **e** Overexpression of HA-TCOF1 in MDA-MB-468 and HCC1806 spheroids. Whole-cell lysates were subjected to immunoblotting. f KIT mRNA levels in MDA-MB-468 spheroids overexpressing HA-TCOF1. Data represent mean ± SEM of three independent experiments, ***p < 0.001. **g** Mammosphere-formation assay of MDA-MB-468 cells upon KIT knockout. Error bars, mean ± SEM of three independent experiments. *p < 0.05. **h**, **i** MDA-MB-468 cells expressing HA-KIT or control vector were infected with tet-on TCOF1 or CTL gRNA. Cells were treated with dox (100 ng/ml) for 5 days and then subjected to mammosphere-formation assay. Error bars, mean ± SEM of three independent experiments. *p < 0.05. Whole-cell lysates were subjected to immunoblotting. **j** Ratio of secondary mammosphere number to primary mammosphere number. Data represents mean ± SEM of three independent experiments. *p < 0.05. Experiments in **c**–**e** were repeated twice independently with similar results. *p* values were calculated by two-sided Student’s *t* test in **b**, **f**, **g**, **i**, **j**.

To explore the molecular mechanisms by which TCOF1 regulates CSCs, we performed RNA-seq to determine transcriptome changes upon TCOF1 knockout. The downregulated genes in TCOF1-knockout MDA-MB-468 mammospheres were shown in the heatmap (Fig. 4a). To prioritise TCOF1 target genes for functional studies, we focused on genes that have been reported to modulate stem cells or CSCs. KIT, FZD8 and NOS2 have been shown to regulate cell differentiation and/or stem cell maintenance [40–44]. In addition, high KIT mRNA expression in basal-like breast cancer is correlated with shorter overall survival [45]. We, therefore, prioritised on testing whether these genes mediate the effect of TCOF1 in TNBC stemness. RT-qPCR confirmed that mRNA levels of KIT, FZD8 and NOS2 were decreased upon TCOF1 knockout (Fig. 4b). Immunoblot analysis shows that TCOF1 depletion led to reduced KIT and FZD8 protein expression in MDA-MB-468 3D spheroids (Fig. 4c). Downregulation of KIT was also observed in two distinct TNBC lines, HCC1806 and HCC1143 (Fig. 4d). Next, we overexpressed HA-TCOF1 in TNBC cells and observed upregulation of KIT protein expression (Fig. 4e) as well as mRNA levels (Fig. 4f). Agreeing with these findings, expression of KIT is positively correlated with TCOF1 expression in a gene expression data set of 55 TNBC samples from lymph node-negative systemically untreated patients (GSE7390; Fig. S5A). The expression of KIT and TCOF1 in normal breast tissues is also positively correlated (GEPIA [46], *n* = 178; Fig. S5B). We then examined the effects of TCOF1 on downstream pathways of KIT, and our results indicated that phosphorylation of KIT, AKT, STAT3 and ERK was inhibited upon TCOF1 knockout (Fig. 4c). Conversely, overexpression of TCOF1 resulted in activation of these known downstream molecules (Fig. 4e), supporting the notion of increased KIT signalling in TCOF1-overexpressed TNBC cells. As the role of KIT in breast CSC properties has not been reported, we first examined whether depleting KIT had any effect on TNBC stemness. Knockout of KIT impaired mammosphere formation as well as self-renewal ability (Fig. 4g). To explore this further and evaluate whether KIT is a downstream target of TCOF1 in CSC maintenance, we overexpressed KIT in rescue experiments. Whereas overexpression of KIT alone had no effect on mammosphere formation, impairment of mammosphere-formation ability by TCOF1 knockout could be rescued by KIT overexpression (Fig. 4h, i). KIT also rescued the ratio of secondary to primary mammosphere numbers (Fig. 4j), indicating that it mediates the function of TCOF1 in self-renewal of CSCs. Interestingly, overexpression of KIT does not rescue the growth of TCOF1-depleted spheroids nor cells cultured in 2D (Fig. S6A), demonstrating the specific role of KIT in mediating TCOF1’s function in CSCs. We have also examined whether FZD8 or NOS2 is a mediator of TCOF1 in regulating CSCs. Overexpression of FZD8 or NOS2 did not rescue mammosphere formation impaired by TCOF1 knockout in TNBC cells (Fig. S6B, C). These findings indicate that KIT, but not FDZ8 or NOS2, plays a crucial role in TCOF1-mediated CSC regulation.

### TCOF1 is regulated by a TNBC-specific super-enhancer

**Fig. 5:**
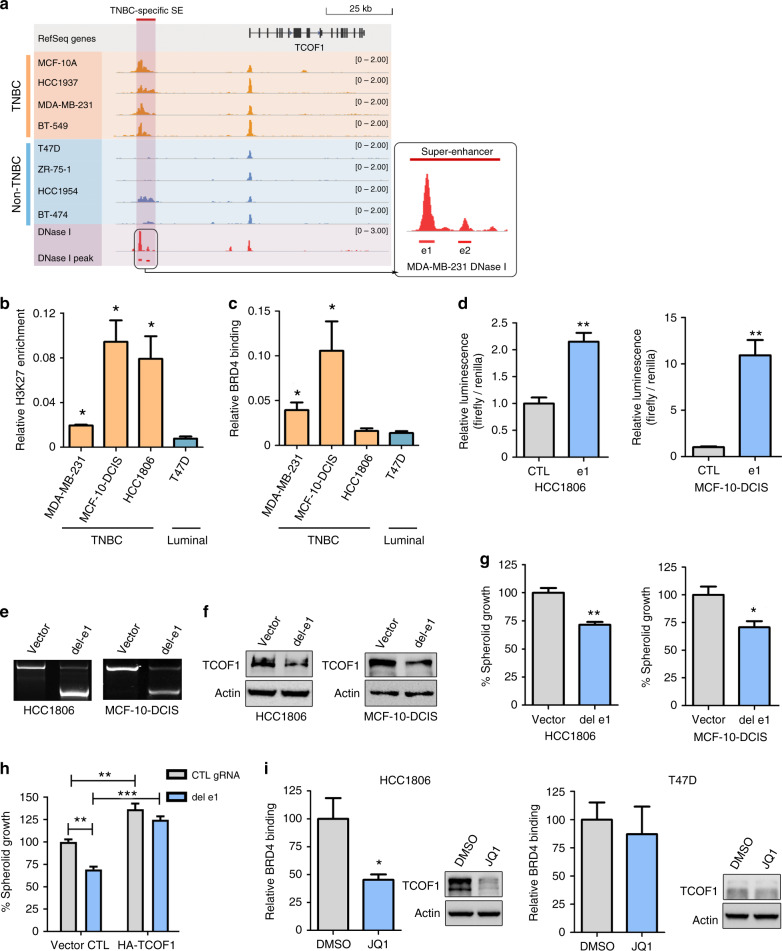
Super-enhancer drives the expression of TCOF1 in TNBC cells. **a** Schematic of the putative super-enhancer of TCOF1, indicated by H3K27ac ChIP-seq analysis. e1 and e2 represent constituent enhancers of the super-enhancer predicted by DNase-I hypersensitive seq analysis in MDA-MB-231 cells. **b** H3K27ac ChIP-qPCR using primers amplifying e1 of TCOF1-associated super-enhancer. Data represent mean ± SEM of three independent experiments. *p < 0.05. **c** BRD4 ChIP-qPCR using primers amplifying e1 of TCOF1-associated super-enhancer. Data represent mean ± SEM of three independent experiments. *p < 0.05. **d** Enhancer activity of e1 in HCC1806 and MCF-10-DCIS cells measured by dual-luciferase reporter assay. Data represent mean ± SEM of three independent experiments. **p < 0.01. **e** Crispr/cas9-mediated DNA deletion of e1 (del-e1) in HCC1806 and MCF-10-DCIS cells. PCR products were analysed by gel electrophoresis. Experiments were repeated twice independently with similar results. **f** Immunoblotting showing TCOF1 expression in cells with or without e1 deletion. Experiments were repeated twice independently with similar results. **g** Spheroid growth of HCC1806 and MCF-10-DCIS cells upon e1 deletion. Data represent mean ± SEM of three independent experiments. *p < 0.05; **p < 0.01. **h** Spheroid growth of HCC1806 with e1 deletion rescued by TCOF1 overexpression. **p < 0.01; ***p < 0.001. **i** HCC1806 and T47D cells treated with JQ1 were subjected to BRD4 ChIP-qPCR and immunoblotting analysis. Data represent mean ± SEM of three independent experiments. *p < 0.05. *p* values were calculated by two-sided Student’s *t* test in **b**–**d**, **g**–**i**.

The regulation of TCOF1 expression is poorly understood. In the context of cancer, our bioinformatics analysis on public data sets uncovered a super-enhancer that potentially drives TCOF1 expression [12]. Figure 5a illustrates the enrichment of acetylation of histone H3 at lysine 27 (H3K27ac) in the super-enhancer SE324 region in TNBC lines compared to non-TNBC lines. We performed ChIP-qPCR on e1 region of SE324, which overlapped with high H3K27ac signals and with the greatest DNase I hypersensitivity in TNBC lines. Our data confirmed that e1 region in TNBC lines was enriched with H3K27ac as well as binding of coactivator BRD4, characteristics of active enhancer (Fig. 5b, c). To determine experimentally whether super-enhancer drives TCOF1 expression, we first performed luciferase reporter assays for the e1 region and showed that the e1 enhancer had significant activities in basal-like HCC1806 and MCF-10-DCIS lines (Fig. 5d), confirming it as a potent regulatory element in the super-enhancer of TCOF1. We then deleted e1 using Crispr/Cas9 system and verified deletion with PCR (Fig. 5e) and DNA sequencing (Fig. S7). Deletion of e1 resulted in marked decrease of TCOF1 expression (Fig. 5f), demonstrating that TCOF1 is a target gene of super-enhancer SE324. Furthermore, deletion of e1 region led to reduction of spheroid growth (Fig. 5g), whereas overexpression of TCOF1 rescued the phenotype (Fig. 5h), indicating the functional importance of TCOF1-associated super-enhancer. JQ1 is a BET inhibitor that shows promising anticancer potential in various cancers, including TNBC [47]. To examine its effect on the binding of BRD4 to TCOF1 super-enhancer and TCOF1 expression, we performed ChIP-qPCR and immunoblotting in JQ1- or dimethyl sulfoxide-treated breast cancer cells. Treatment of TNBC cells with JQ1 potently reduced the binding of BRD4 to the super-enhancer as well as TCOF1 protein levels (Fig. 5i). In contrast, JQ1 had no effect on BRD4 binding or TCOF1 expression in luminal cells (Fig. 5i). Finally, we explored potential TFs binding to the e1 region of SE324, by obtaining known TF-binding sites from JASPAR database (http://jaspar.genereg.net/) [33] as well as interrogating the nucleosome-free regions using DNase-seq data. One hundred and twenty-seven candidate TFs binding to e1 were identified (Table S4). Of these candidates, YY1 and c-MYB have been shown to bind to the promoter of TCOF1 [48, 49], whereas PLAG1 and FOSL1 were demonstrated to regulate TNBC enhancer activities [50]. These four candidates will be prioritised for examining their function in mediating TCOF1 overexpression via super-enhancer in future studies. Taken together, our findings uncovered TCOF1 as a novel oncogenic gene regulated by a TNBC-specific super-enhancer.

## Discussion

Despite major improvements made with diagnosis and treatment strategies for luminal and HER2-overexpressed tumours, prognosis of TNBC patients remains poor. We therefore sought to discover novel genes and pathways driving TNBC stemness, which is an exciting area of investigation with potential of yielding next-generation therapeutic strategies. Indeed, several therapies targeting CSC-associated signal pathways and microenvironment are already undergoing clinical trials [51]. By performing integrative epigenomic and transcriptomic analyses, we recently demonstrated an important role of super-enhancers in characterising breast cancer subtype-specific identity [12]. We further identified corresponding targeting genes of TNBC-specific super-enhancers, including FOXC1, MET and ANLN, whose function and clinical relevance in breast cancer have been well documented [52–55]. In this study, we leverage the multiomic analysis and report the identification of TCOF1 whose expression is upregulated in 32% of TNBC. Additional mining of METABRIC data revealed that high expression of TCOF1 is correlated with poor survival of basal-like, TNBC patients. TCOF1 has been shown to play critical roles in several biological processes, such as neural crest formation and development [56], regulation of translation [16] and human craniofacial development [57]. Yet, the disease-associated overexpression and function of TCOF1 in human cancers have never been characterised. One of the most important findings here is that we identified TCOF1 as a functionally significant gene that promotes growth and CSC properties of TNBC. We showed that alteration of TCOF1 levels in TNBC cells affected their tumour-initiating capacity in vivo. Moreover, knockout of TCOF1 significantly inhibited growth of TNBC spheroids in 3D culture, without affecting apoptotic cell death. Interestingly, knockout of TCOF1 resulted in increased cell death in mammospheres. Whether TCOF1 specifically modulates death of CSCs await further investigation.

Under the Lehmann classification, TNBC can be divided into three main groups: basal-like, mesenchymal-like, and LAR, which are addicted to different signalling pathways and display differential sensitivity to therapeutic agents [7]. Notably, whereas TCOF1 plays a critical role in spheroid growth of the basal-like subtype, our data suggest that modulating the expression of TCOF1 has no effect on the growth of mesenchymal-like spheroids. According to Lehmann classification [7], basal-like subtype can further be divided into BL1 and BL2, whereas mesenchymal-like subtype can further be divided into MSL and M. In our study, we have examined the function of TCOF1 in BL1 (MDA-MB-468, HCC1937, HCC1143), BL2 (HCC1806), M (BT549) and MSL (Hs578T, MDA-MB-436 and MDA-MB-231) subtypes. It is worthwhile to mention that, in another classification scheme, namely, Neve classification, breast tumour lines are divided into Basal A, Basal B, and Luminal [58]. BL1 and BL2 of Lehmann classification belong to Basal A of Neve classification, whereas M and MSL belong to Basal B. In line with our data, results from a whole-genome small hairpin RNA screen demonstrated that TCOF1 is a preferentially essential gene in Basal A but not other subtypes of breast cancer in the Neve classification [59]. Interestingly, TCOF1 has been shown to mediate DNA damage response in neuroepithelial cells, where it cooperates with ATM and NBS1 to maintain genomic integrity upon DNA damage, which in turn promotes cellular resistance to DNA-damaging agents [18, 60]. Our data of combinatorial effects of TCOF1 depletion and chemotherapeutic agents on TNBC growth are in line with the potential role of TCOF1 in DNA damage response, as chemotherapeutic drugs including Paclitaxel and Cisplatin can induce cellular DNA damage. Another potential reason for the combinatorial effects is that basal-like breast tumours are enriched in genes regulating cell proliferation [7], which could result in the enhancement of antitumour effects by chemotherapeutic agents upon TCOF1 knockout. It would be interesting to test whether these mechanisms are responsible for the specific effect of TCOF1 on basal-like TNBC. Recently, Wrenn et al. showed that BL2 but not mesenchymal-like TNBC contains nanolumina enriched in the growth factor epigen, which plays a critical role in collective metastasis [61]. These findings not only point to the importance of identifying distinct drivers for different subtypes of TNBC but also suggest that overexpression of TCOF1 in basal-like TNBC could be used as a patient-tailoring strategy for therapeutic benefit. Targeted protein degradation using proteolysis targeting Chimeras (PROTACs), a recently emerged therapeutic modality in drug discovery, could be explored for developing therapeutic strategy against undruggable proteins including TCOF1. Other potential approaches for targeting TCOF1 include the use of Crispr/Cas9 and RNA interference technologies and development of compounds covalently crosslinked to specific RNA [62], as well as innovative strategies to screen small-molecule inhibitors for proteins considered to be undruggable [63].

Mechanistically, our RNA-seq data showed that TCOF1 positively regulates the expression of several stem cell regulators, including KIT, FDZ8 and NOS2. KIT, a tyrosine-protein kinase that acts as a cell-surface receptor for the stem cell factor, has been established as a central regulator of normal stem cell properties [40, 64]. In the context of human cancer, ovarian and osteosarcoma cancer cells expressing KIT were reported to be chemotherapy resistant and have increased tumorigenicity [65–67]. In addition, activation of KIT promotes ovarian CSC survival and self-renewal [41, 68], indicating that KIT regulates not only normal stem cells but also CSCs of certain tumour types. IHC analysis has revealed that KIT is expressed in 45% of TNBCs [69, 70]. Higher expression of KIT in TNBC is associated with larger tumours and lymphovascular invasion [70]. Moreover, increased expression of KIT was reported to be more common in basal-like breast cancer and was correlated with poor patient survival [45]. Whether KIT plays a role in breast CSC regulation, however, remains elusive. In TNBC, we found that impaired mammosphere formation of TCOF1-knockout cells can be rescued by KIT, indicating that KIT is a downstream effector of TCOF1 in mediating CSC self-renewal ability. These data agree with the inhibition of signalling pathways downstream of KIT in TCOF1-depleted cells. Indeed, STAT3 and Akt/β-catenin pathways have been shown to play an important role in driving the enrichment of breast CSCs [39, 71], and β-catenin has been demonstrated to mediate the effect of KIT on promoting stemness of ovarian cancer [68]. Interestingly, impairment of cell proliferation in 2D and 3D by TCOF1 knockout could not be rescued by KIT overexpression, indicating that another downstream effector is responsible for mediating TCOF1’s function in cell proliferation and/or survival of bulk tumour cells. Downregulated genes in TCOF1-knockout cells from RNA-seq that have cell proliferation-related function will be prioritised for spheroid rescue study in the future.

The functions of TCOF1 in ribosome biogenesis and RNA polymerase I (Pol I) regulation are well established. In mouse embryos, it has been shown that TCOF1 can interact with upstream binding factor (UBF) to promote pre-rRNA expression [13]. Furthermore, TCOF1 can bind to rDNA promoter and recruit RNA Pol I to nucleolus in a UBF-independent manner [72]. Diminishment of rRNA integrity, indicated by the Y10B antibody staining, was also observed in TCOF+/− mouse embryos [56]. In our study, knockout of TCOF1 affects neither the expression levels of 45S rRNA or 5S rRNA nor the integrity of rRNA in TNBC cells. These observations suggest that the regulation of rDNA transcription by TCOF1 is context dependent. Our data indeed are in line with studies in TCOF1+/− embryos, where inhibiting p53 rescued the craniofacial anomality of TCS without restoring ribosome biogenesis, implying that TCOF1 has molecular functions distinct from the regulation of translation [73]. The precise mechanism by which TCOF1 regulates KIT expression remains to be determined. The major function of TCOF1 has been attributed to its role in the nucleolus. A recent high-throughput proteomic study, however, demonstrated an interaction between TCOF1 and the RNA Polymerase II initiation factor TFIID, which acts as a central regulator for transcription initiation [74, 75]. TFIID has been shown to play an important role in supporting pluripotency of ovarian CSCs, which are enriched in KIT. Interestingly, a structurally and functionally related protein of TCOF1, NOLC1, has also been demonstrated to act as a TF, by interacting with TFIIB to activate transcription of alpha-1 acid glycoprotein [76]. Whether TCOF1 functions as a co-factor to enhance KIT transcription awaits further investigation.

The expression and regulation of TCOF1 has not been well studied. Most of the work has been done in neuroepithelium, where TCOF1 is shown to express strongly in neural crest progenitor cells of mouse embryos [56]. In TCS, frameshift deletions or duplications of base pairs of TCOF1 gene results in premature termination codons and mRNA degradation [77]. Post-translationally, TCOF1 has been reported to be monoubiquitylated by KBTBD8 in human embryonic stem cells, and it is postulated that TCOF1 and KBTBD8 together play an important role in determining cell fate by producing ribosomes with distinct translational output [16]. In our study, despite overexpression of TCOF1 was found in 32% of TNBC, no copy number alteration of TCOF1 gene region is observed in our genomic analysis. Instead, we discovered a TNBC-specific super-enhancer (SE324), which drives TCOF1 expression. When we performed the super-enhancer/gene expression analyses, we looked for TNBC-specific super-enhancers 500 kb upstream of genes that are upregulated in TNBC samples. Although we cannot preclude the possibility of recruitment of far-away genes to SE324, it is very likely that TCOF1 is the sole gene target of this super-enhancer. Regulation of genes by super-enhancers in breast cancer have not been well studied. Nevertheless, CD47 has been demonstrated to be upregulated in breast cancer by super-enhancer [78]. In addition, CDK7 inhibitors can inhibit the growth of patient-derived breast xenograft and potentially act upon the TNBC-specific super-enhancers, which regulate certain key oncogenes [79]. A computational pipeline has recently been developed to identify transcribed enhancers in breast cancer and demonstrated that TFs such as FOSL1 and PLAG1 play key roles in regulating the activities of TNBC enhancers [50]. In addition, a few TFs, including YY1, Cebpb, Zfp161, Sp1 and c-MYB, have been described to bind to TCOF1 promoter and modulate its expression [48, 49]. Our bioinformatic analysis on TF binding predicts that FOSL1, PLAG1, YY1 and c-MYB bind to e1 region of SE324 in TNBC cells. It would be interesting to examine whether these TF candidates mediate the super-enhancer-driven overexpression of TCOF1 and their functions in TNBC, as a potential strategy for targeting TCOF1 therapeutically. In summary, we leveraged the multiomic profiling on super-enhancers to uncover a novel oncogenic gene, TCOF1, in modulating CSC properties of breast cancer. Considering the clinical significance of TCOF1 in TNBC and its minimal expression in normal breast tissues, there may be therapeutic opportunity of targeting TCOF1 for patients harbouring TCOF1-high-expressing basal-like TNBC.

## Supplementary information

Supplementary information

Supplementary table 1

Supplementary table 2

Supplementary table 3

Supplementary table 4

## Data Availability

Previously published data sets are available in METABRIC Data set ID: EGAD00010000210 (https://ega-archive.org/studies/EGAS00000000083). RNA sequencing data generated from this study have been deposited in GEO under accession code GSE168835.
