# Supplementary material for: TCOF1 upregulation in triple-negative breast cancer promotes stemness and tumour growth and correlates with poor prognosis
Source: Br J Cancer. 2021 Oct 30;126(1):57–71. doi: 10.1038/s41416-021-01596-3 (PMC8727631; doi:10.1038/s41416-021-01596-3)
Supplement: Supplementary file 1 — Supplementary information [file 41416_2021_1596_MOESM1_ESM.docx]

**SUPPLEMENTARY INFORMATION**

**TCOF1 Upregulation in Triple-negative Breast Cancer Promotes Stemness and TUmor Growth, and Correlates with Poor prognosis**

**
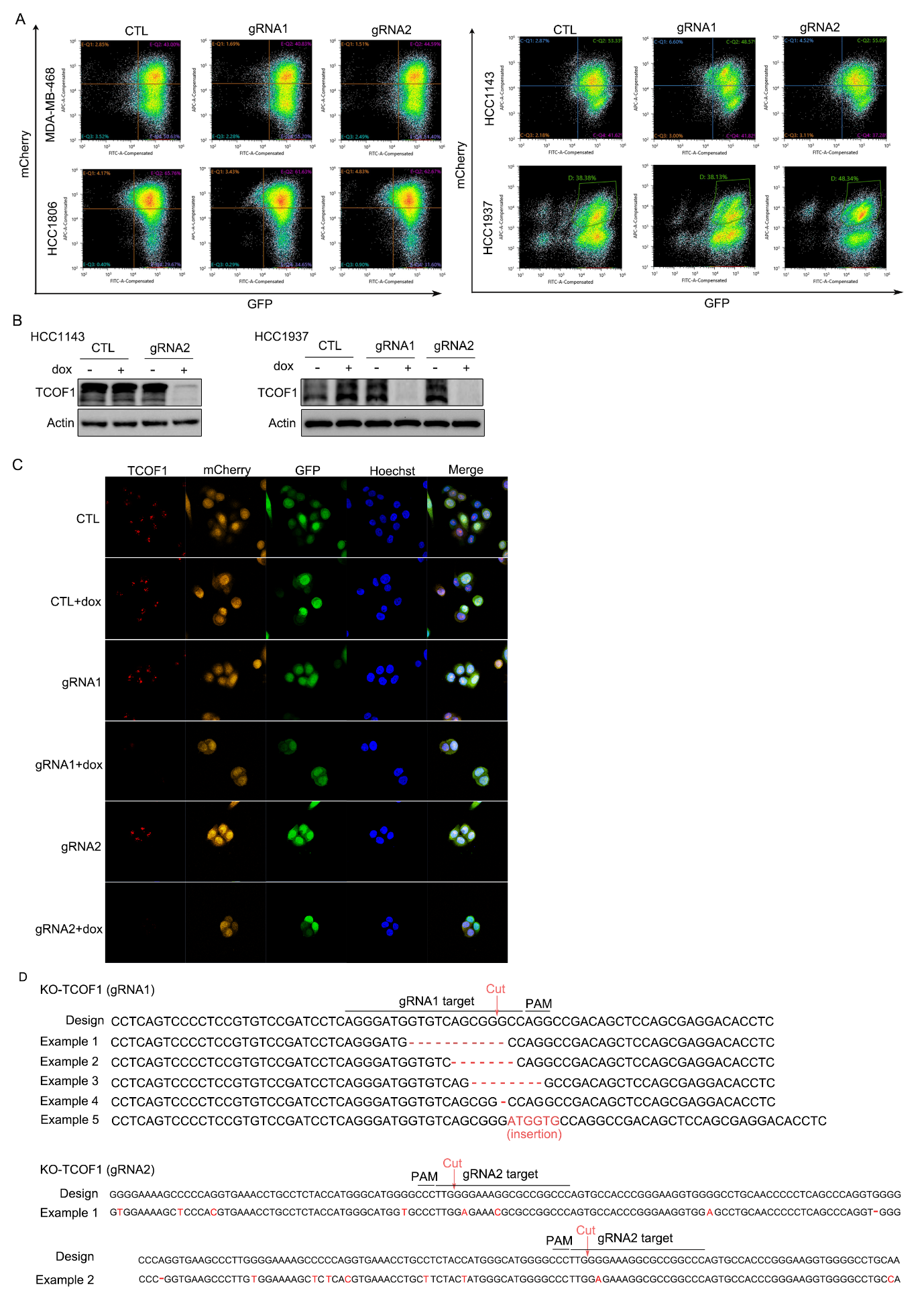
**

**Fig. S1 Crispr/cas9 mediated knockout of TCOF1**

**(A)** TNBC lines MDA-MB-468, HCC1806, HCC1143 and HCC1937 were infected with lentiviral vectors carrying spCas9 (co-expressed with mCherry) and inducible sgRNA (co-expressed eGFP). Cells expressing high levels of mCherry and eGFP were sorted by FACS. **(B)** HCC1143 and HCC1937 cells expressing tet-on TCOF1 gRNA or vector control (CTL) were treated with doxycycline (dox; 100 ng/ml) for 5 days. Whole-cell lysates were subjected to immunoblotting. Experiments were repeated twice independently with similar results. **(C)** Immunofluorescence was performed on MDA-MB-468 cells with or without TCOF1 knockout. Experiments were repeated twice independently with similar results. **(D)** PCR products of genomic DNA from MDA-MB-468 with TCOF1 knockout were cloned into individual vectors and sequenced. Sequencing results represent the mutations induced by gRNA1 and gRNA2.


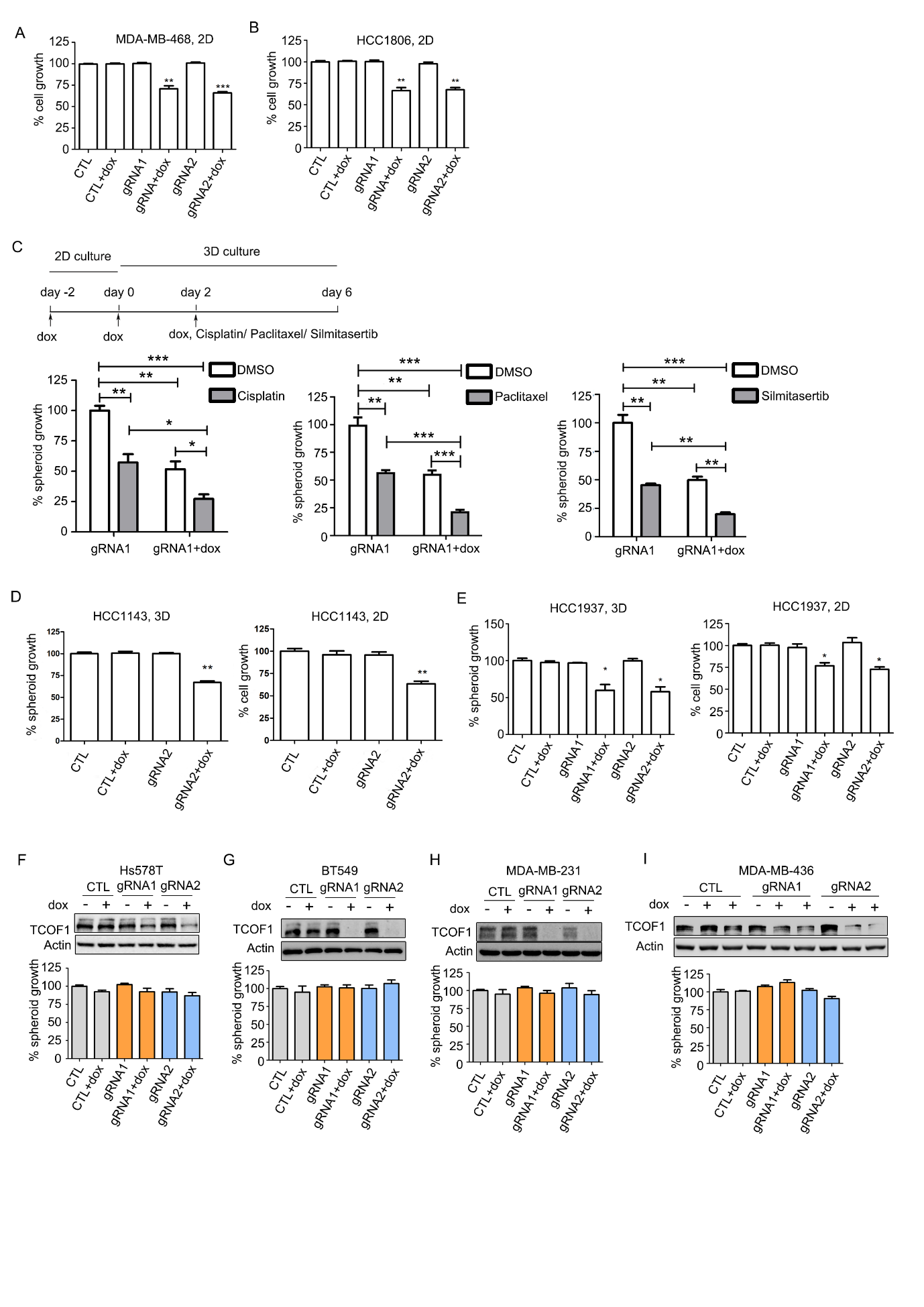


**Fig. S2 Knockout of TCOF1 led to growth inhibitory effect on basal-like TNBC**

**(A, B)** Viability of MDA-MB-468 and HCC1806 cells with or without TCOF1 knockout (2D culture). Data represent mean ± SEM of 3 independent experiments. **, *p* < 0.01; ***, *p* < 0.001. **(C)** Schematic of TCOF1 knockout combined with Cisplatin (1.25 µM), Paclitaxel (3.5 nM) or Silmitasertib (5 µM) treatment on HCC1806 cells. Bar graph depicts the percentage of spheroid growth. Data represent mean ± SEM of 3 independent experiments. *, *p* < 0.05. **，*p* < 0.01; ***，*p* < 0.001. **(D, E)** Bar graphs depict growth of HCC1143 and HCC1937 cells with or without TCOF1 knockout (3D spheroids and 2D culture). Error bars, mean ± SEM of 3 independent experiments. *, *p* < 0.05; **, *p* < 0.01. **(F-I)** Bar graphs depict growth of Hs578T, BT549, MDA-MB-231 and MDA-MB-436 spheroids with or without TCOF1 knockout. Error bars, mean ± SEM of 3 independent experiments. Whole-cell lysates were subjected to immunoblotting. P-values were calculated by two-sided Student's t- test in (A-E).


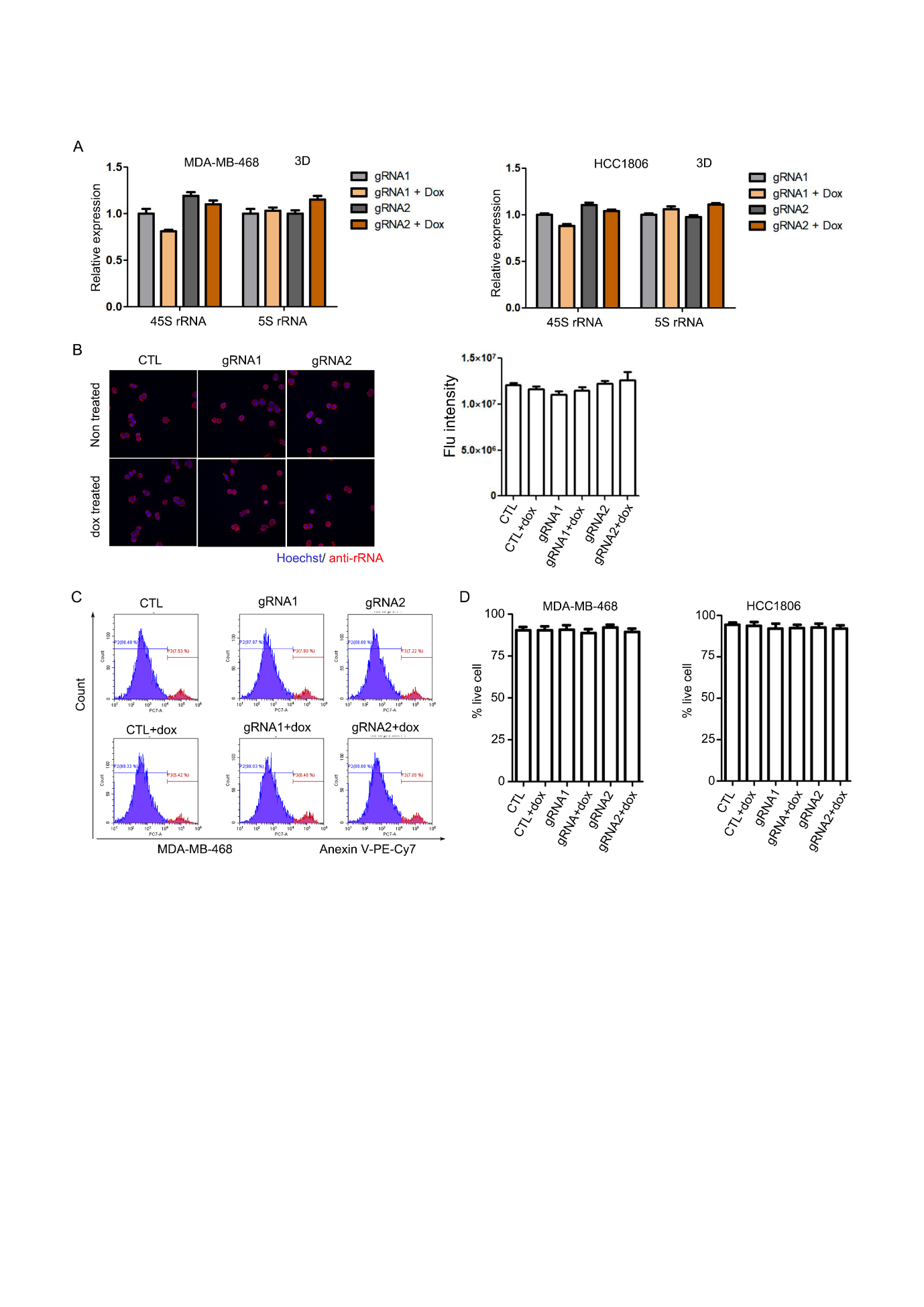


**Fig. S3 Knockout of TCOF1 did not impair rRNA integrity**

**(A)** RT-qPCR detection of 45S rRNA and 5S rRNA of MDA-MB-468 and HCC1806 spheroids with or without TCOF1. Experiments were repeated twice independently with similar results. **(B)** Immunofluorescence was performed on MDA-MB-468 cells with or without TCOF1 knockout. rRNA were stained with Y10b antibody. Representative images captured by High-content screening/analysis (HCS) platform are shown. Bar graphs depict the signals quantified by HCS Studio software. Data represent mean ± SEM of 3 independent experiments. **(C)** MDA-MB-468 spheroids with and without TCOF1 knockout were stained with Annexin V, followed by flow cytometry analysis. Experiments were repeated twice independently with similar results. **(D)** Trypan blue assay were performed on MDA-MB-468 and HCC1806 cells with or without TCOF1 knockout. Bar graphs depict the percentage of live cells.


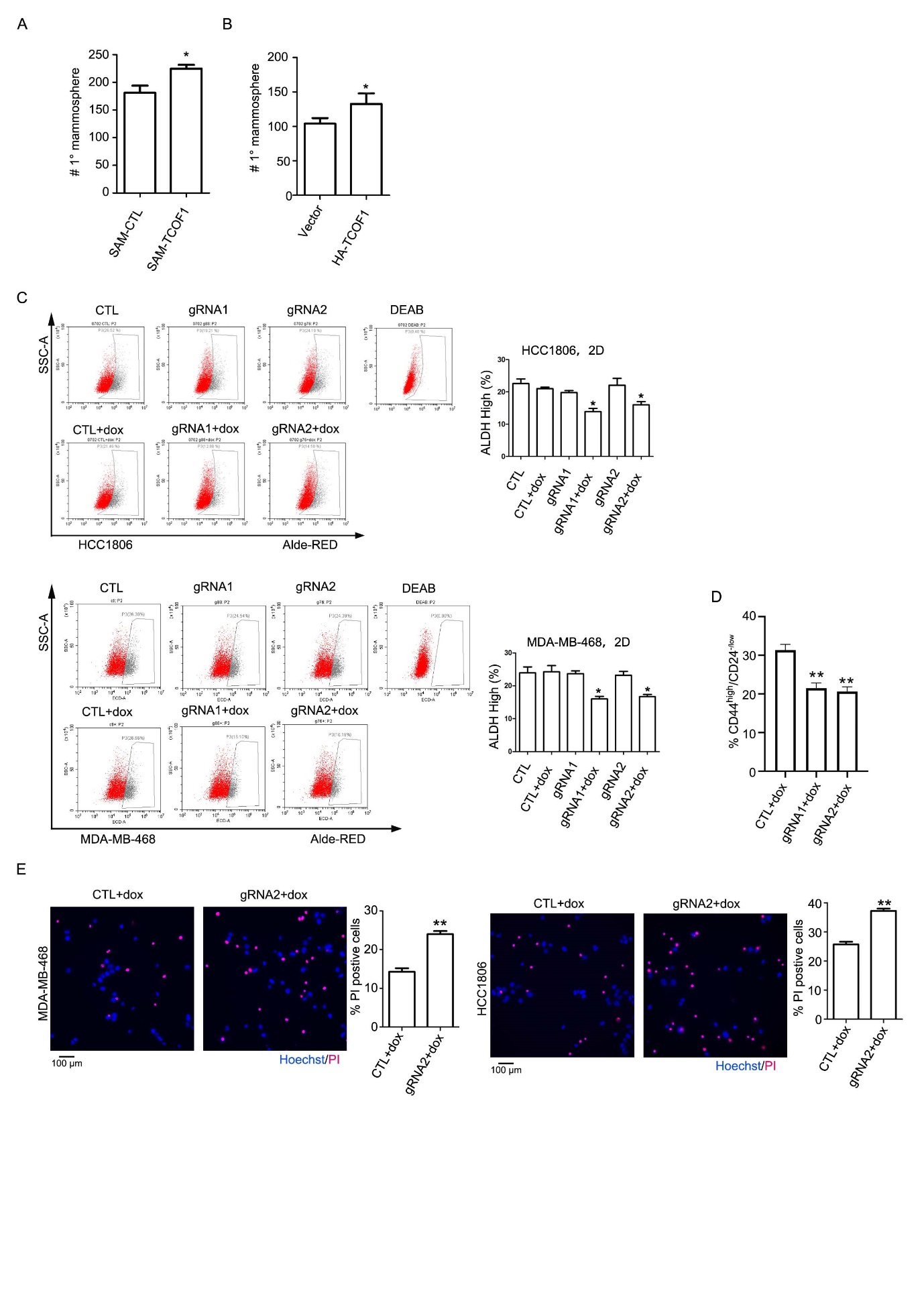


**Fig. S4 Knockout of TCOF1 led to decreased population of TNBC CSCs**

**(A)** MDA-MB-468 cells with or without overexpression of endogenous TCOF1 by CRISPR/Cas9 Synergistic Activation Mediator (SAM) technique were subjected to mammosphere formation assay. Error bars, mean ± SEM of 3 independent experiments. *, *p* < 0.05. **(B)** MDA-MB-468 cells with or without overexpression of exogenous HA-TCOF1 were subjected to mammosphere formation assay. Error bars, mean ± SEM of 3 independent experiments. *, *p* < 0.05. **(C)** ALDH activity of HCC1806 and MDA-MB-468 cells with or without TCOF1 knockout (2D culture), measured by AldeRed ALDH detection assay. Bar graphs depict the percentage of ALDH high cells. Data represent mean ± SEM of 3 independent experiments. *, *p* < 0.05. **(D)** Population of CD44^high^/CD24^-/low^ of HCC1806 cells from 3D cultures detected by flow cytometry. Error bars, mean ± SEM of 3 independent experiments. **, *p* < 0.01. **(E)** PI staining of cells in MDA-MB-468 and HCC1806 mammospheres. Representative images are shown. Bar graph depicts the percentage of PI positive cells (dead cells). Data represent mean ± SEM of 3 wells. 25 fields of images were captured for each well. **, *p* < 0.01. P-values were calculated by two-sided Student's t- test in (A-E).

**
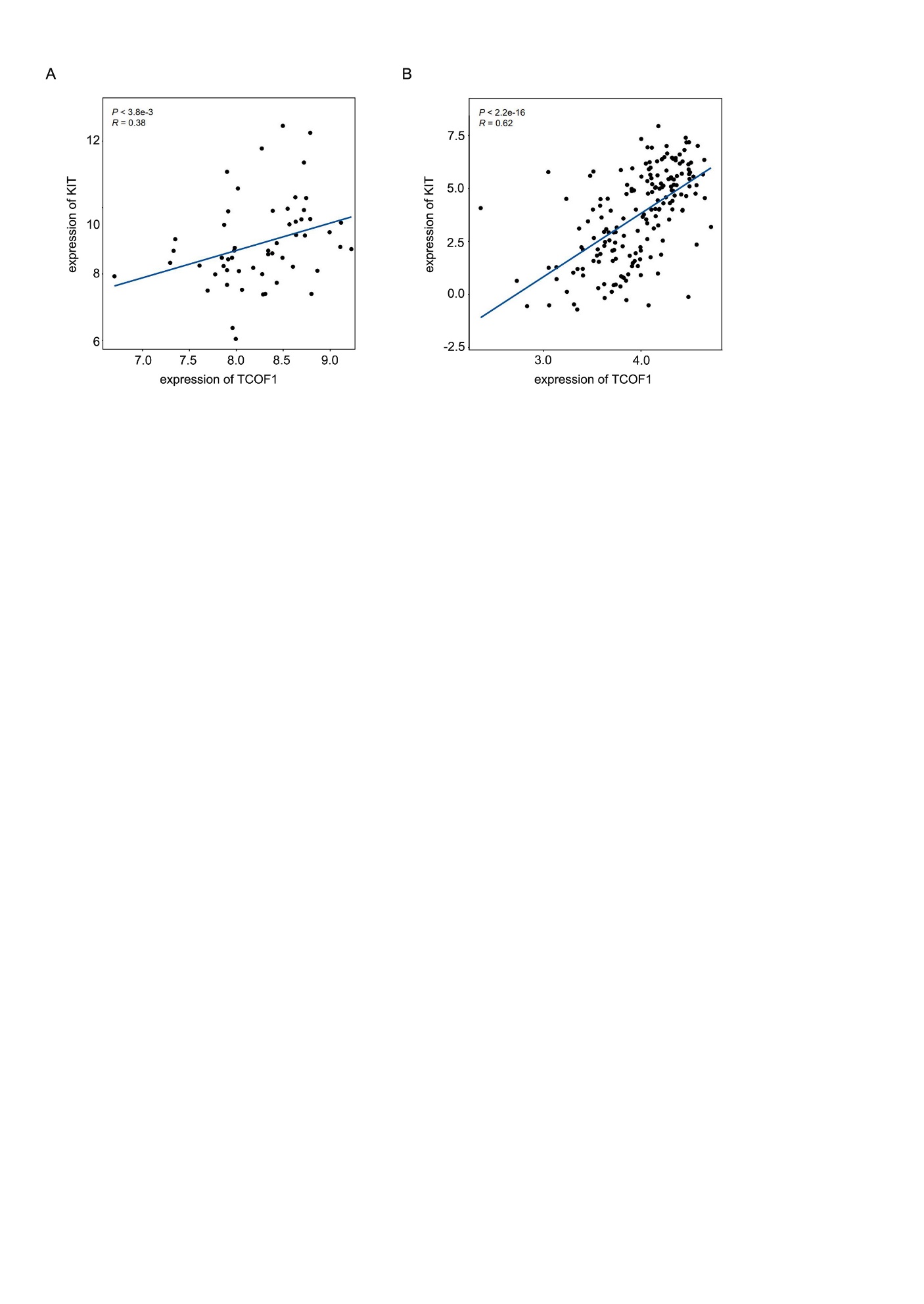
**

**Fig. S5 Positive correlation between mRNA levels of TCOF1 and KIT.**

**(A)** mRNA levels of TCOF1 and KIT are positively correlated in 55 TNBC patient samples (GSE7390 dataset). **(B)** mRNA levels of TCOF1 and KIT are positively correlated in 178 normal mammary tissues (GTEx dataset). R represents Spearman correlation coefficient. Trendline is determined by linear regression.


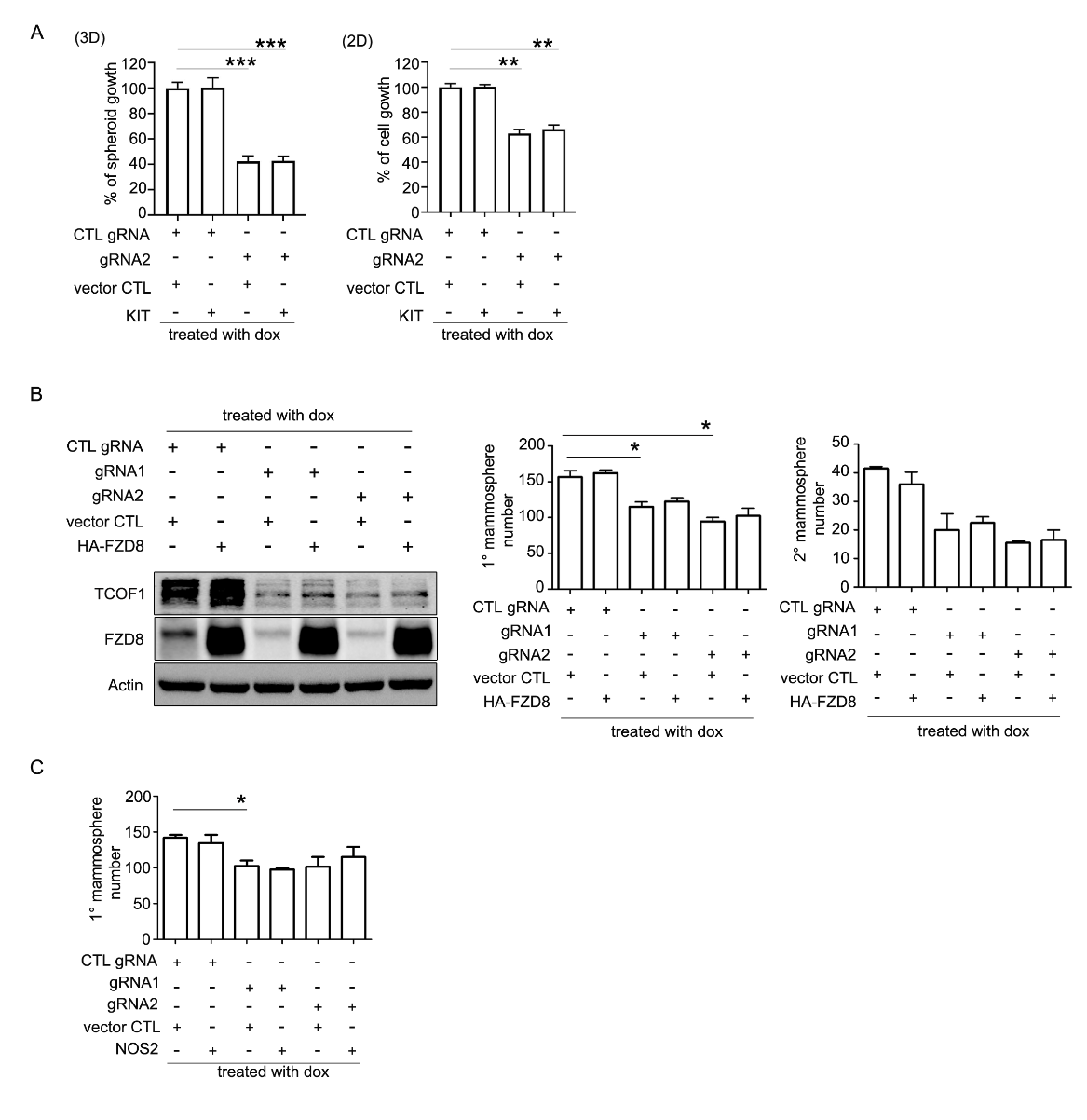


**Fig. S6 Overexpression of FZD8 or NOS2 did not rescue mammosphere formation impaired by TCOF1 knockout.**

**(A)** Bar graphs depict growth of HCC1806 cells expressing HA-KIT or control vector which were with or without TCOF1 knockout (3D spheroids and 2D culture). Error bars, mean ± SEM of 3 independent experiments. ***, *p* < 0.001; **, *p* < 0.01. **(B)** MDA-MB-468 cells expressing HA-FZD8 or control vector were infected with tet-on TCOF1 or CTL gRNA. Cells were treated with dox (100 ng/ml) for 5 days, and then subjected to mammosphere formation assay. Error bars, mean ± SEM of 3 independent experiments. *, *p* < 0.05. Whole-cell lysates were subjected to immunoblotting. **(C)** MDA-MB-468 cells expressing NOS2 or control vector were infected with tet-on TCOF1 or CTL gRNA. Cells were treated with dox (100 ng/ml) for 5 days, and then subjected to mammosphere formation assay. Error bars, mean ± SEM of 3 independent experiments. *, *p* < 0.05. P-values were calculated by two-sided Student's t- test in (A-C).


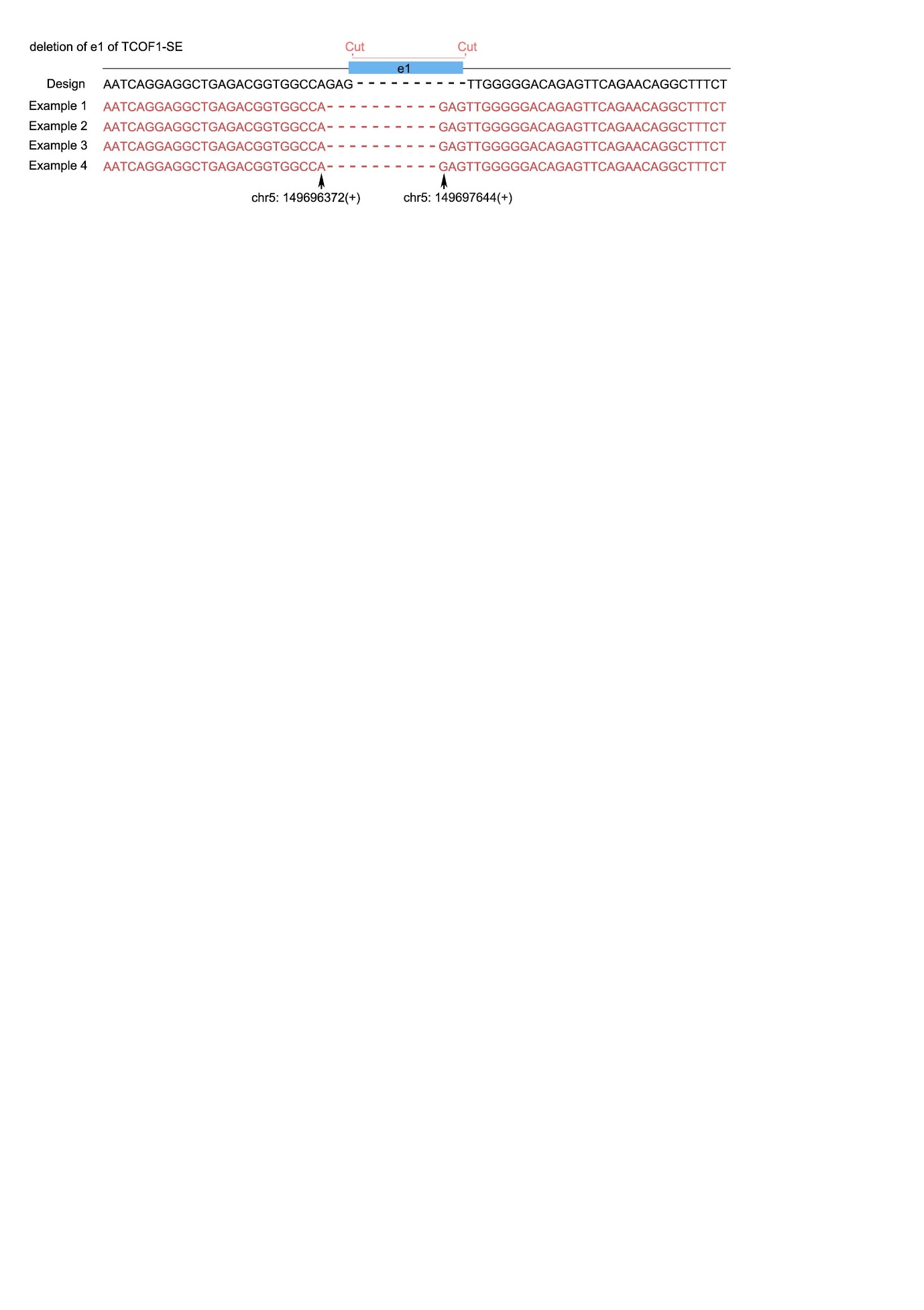


**Fig. S7 Crispr/cas9 mediated deletion of e1 of TCOF1-super-enhancer**

PCR products of genomic DNA from HCC1806 with e1 deletion were cloned into individual vectors and sequenced. Sequencing results represent the deletions induced by Crispr/Cas9-medated DNA deletion.
