# Supplementary material for: TCOF1 upregulation in triple-negative breast cancer promotes stemness and tumour growth and correlates with poor prognosis
Source: Br J Cancer. 2021 Oct 30;126(1):57–71. doi: 10.1038/s41416-021-01596-3 (PMC8727631; doi:10.1038/s41416-021-01596-3)
Supplement: Supplementary file 3 — Supplementary table 2 [file 41416_2021_1596_MOESM3_ESM.docx]

| **Table S2**. Relationship Between TCOF1 Expression and Clinical Features in 959 Breast Cancer Cases. | | | | | |
| --- | --- | --- | --- | --- | --- |
| Variable | Low expression (n=735) | Percent | High Expression (n=224) | Percent | *P* |
| Mean age (years ± SD) | 60.74±13.00 |  | 60.38±12.74 |  | 0.71 |
| Grade |  |  |  |  |  |
| 1 | 61 | 8.31 | 7 | 3.13 | <0.001 |
| 2 | 322 | 43.81 | 78 | 34.82 |  |
| 3 | 352 | 47.89 | 139 | 62.05 |  |
| TNM |  |  |  |  |  |
| 0 | 0 | 0 | 1 | 0.49 | <0.001 |
| I | 255 | 36.85 | 45 | 22.06 |  |
| II | 378 | 54.62 | 138 | 67.65 |  |
| III | 54 | 7.80 | 17 | 8.33 |  |
| IV | 5 | 0.72 | 3 | 1.47 |  |
| Size |  |  |  |  |  |
| <5cm | 338 | 45.99 | 79 | 35.27 | 0.24 |
| >5cm | 397 | 57.37 | 145 | 71.43 |  |
