# Supplementary figures and images for: TCOF1 upregulation in triple-negative breast cancer promotes stemness and tumour growth and correlates with poor prognosis
Source: Br J Cancer. 2021 Oct 30;126(1):57–71. doi: 10.1038/s41416-021-01596-3 (PMC8727631; doi:10.1038/s41416-021-01596-3)

**Table S3. Multivariate Cox regression analyses of potential poor prognostic factors in TNBC**


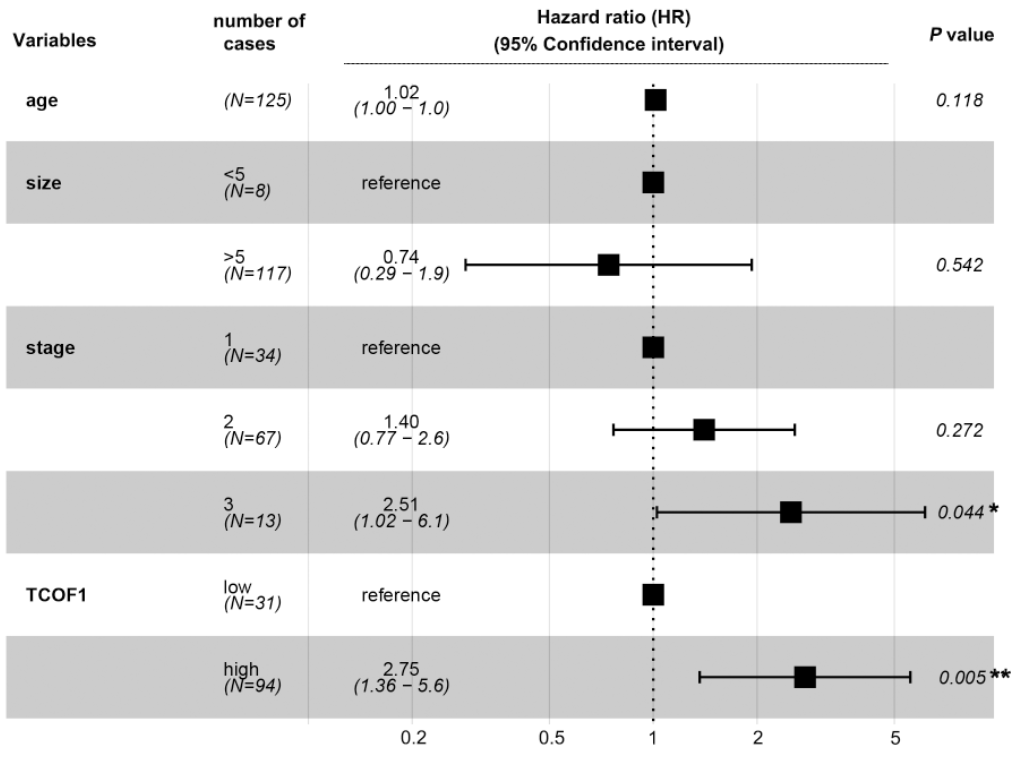

Supplement: Supplementary file 4 — Supplementary table 3 [file 41416_2021_1596_MOESM4_ESM.docx]
